# Shining light on halogen-bonding complexes: a catalyst-free activation mode of carbon–halogen bonds for the generation of carbon-centered radicals

**DOI:** 10.1039/d3sc01724a

**Published:** 2023-05-11

**Authors:** Helena F. Piedra, Carlos Valdés, Manuel Plaza

**Affiliations:** a Departamento de Química Orgánica e Inorgánica, Instituto Universitario de Química Organometálica “Enrique Moles” and Centro de Innovación en Química Avanzada (ORFEO-CINQA), Universidad de Oviedo Julián Clavería 8 33006 Oviedo Spain plazamanuel@uniovi.es

## Abstract

The discovery of new activation modes for the creation of carbon-centered radicals is a task of great interest in organic chemistry. Classical activation modes for the generation of highly reactive radical carbon-centered intermediates typically relied on thermal activation of radical initiators or irradiation with unsafe energetic UV light of adequate reaction precursors. In recent years, photoredox chemistry has emerged as a leading strategy towards the catalytic generation of C-centered radicals, which enabled their participation in novel synthetic organic transformations which is otherwise very challenging or even impossible to take place. As an alternative to these activation modes for the generation of C-centered radicals, the pursuit of greener, visible-light initiated reactions that do not necessitate a photoredox/metal catalyst has recently caught the attention of chemists. In this review, we covered recent transformations, which rely on photoactivation with low-energy light of a class of EDA complexes, known as halogen-bonding adducts, for the creation of C-centered radicals.

## Abstract 2

This review provides a comprehensive overview on examples of how photochemical excitation of halogen-bonding complexes can be useful for the generation of carbon-centered radicals and their participation in synthetic organic transformations.

## Graphical abstract

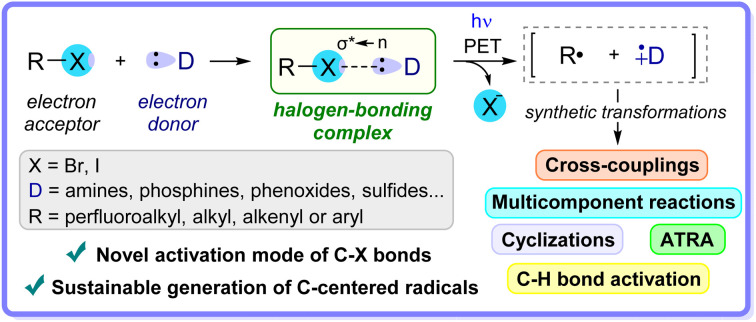

## 1 General introduction

Carbon-centered radicals stand as very useful highly reactive intermediates in synthetic organic chemistry for the construction of high-added-value molecules.^1^ These intermediates are attractive due to the different chemical reactivity and selectivity that they present in comparison to other carbon-centered reactive intermediates carbanions, carbocations or carbenes.^2^ It is worth noting that the unique reactivity that radicals display is dictated by the bond dissociation energy (BDE) and polarity of the reaction coupling partners.^3^ In general, both carbon-centered radicals that are kinetically or thermodynamically stabilized and those that are rather short-lived (*τ* < 1 ms) present long enough lifetimes that enable their utility in synthetic transformations.^4^

**Scheme 1:**
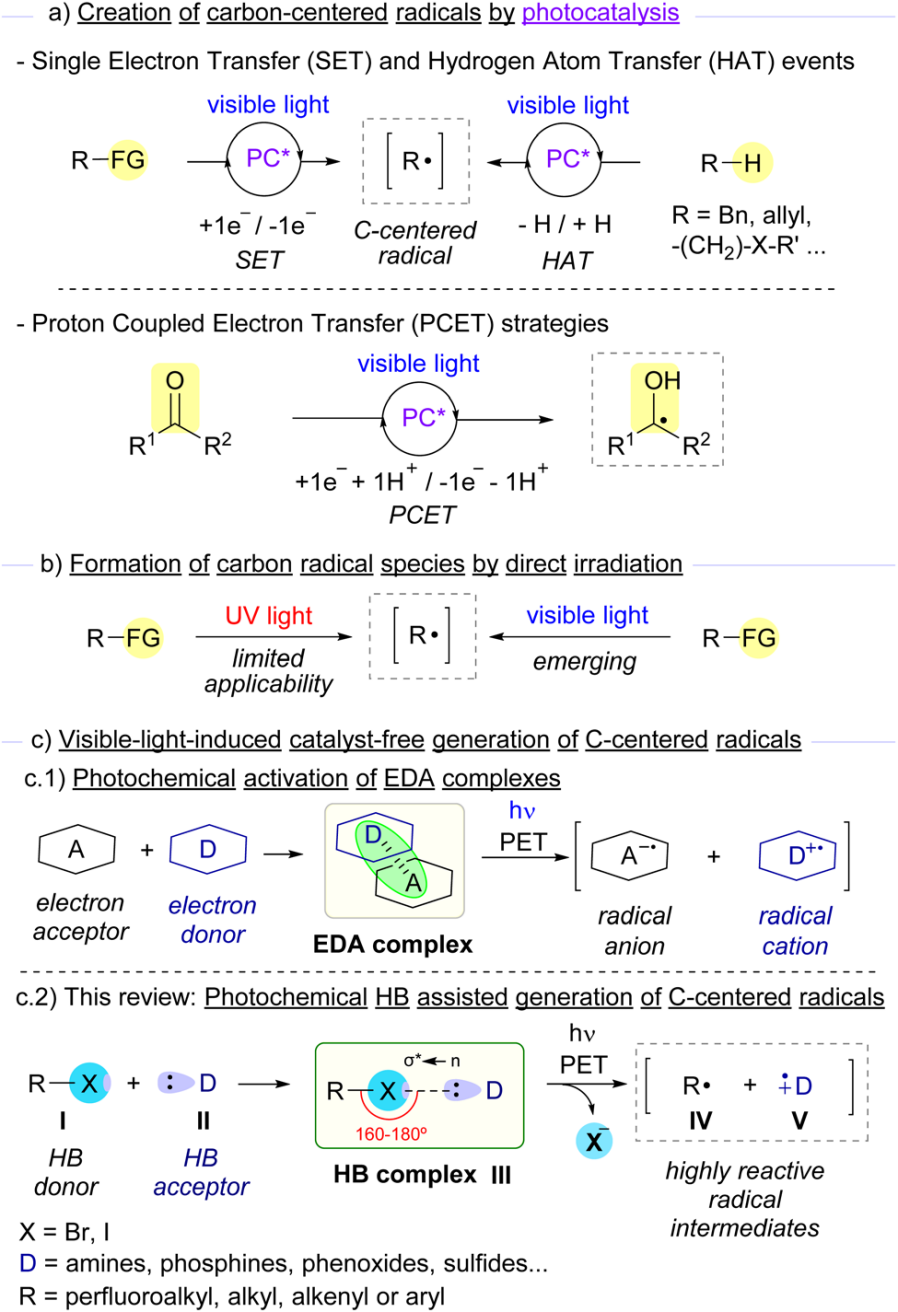
Overview of photochemical access to carbon-centered radicals and aim of this review. FG: functional group.

The first reports on the employment of carbon-centered radical intermediates date back to the 19th century, and since then, a number of synthetically very relevant reactions have been devised, such as several classic name reactions as Birch reduction, Kharasch reaction (also known as Atom Transfer Radical Addition, ATRA), Giese reaction, Minisci reaction, Kolbe electrocoupling, Barton deoxygenation and decarboxylation and pinacol coupling to name a few.^5,6^ Nevertheless, their employment in conventional synthesis has historically been comparatively less employed than polar and pericyclic reactions. Over the past decade, the rapid growth of photoredox chemistry has opened up new avenues for the catalytic generation of carbon-centered radicals, enabling novel organic transformations that otherwise would be challenging, if not impossible, to occur.^7^ Additionally, the development of methods that combine photo- and transition-metal catalysis in a cooperative manner, also known as metallophotoredox, has experienced a tremendous growth in the last few years.^8^ As pointed out before, radical chemistry enabled by photoredox catalysis has been the object of study by many research groups in the last decade.^9^ In these transformations, a photocatalyst (PC) is excited through irradiation with either visible light or low-energy UV light. After irradiation at an appropriate wavelength, the excited state of the photocatalyst (PC*) is transiently reached, which is able to activate appropriate organic substrates to *in situ* generate highly reactive radical intermediates. In the most common activation mode in photoredox catalysis, the excited photocatalyst (PC*) causes the reduction or oxidation of selected photosubstrates by single electron transfer (SET). This event is dictated by a match in the redox potentials of both the catalyst and the substrate, which will eventually result in an oxidative or reductive quenching of the photocatalytic cycle. Different functionalized synthetic precursors such as redox active esters, oxalates, diazonium salts, and others can be employed aiming at the formation of carbon-centered radicals (Scheme 1a).^10^ Moreover, at present, a wide repertoire of photocatalysts is available, which span from the widely used Ir- and Ru-based complexes,^11^ to organophotocatalysts such as acridinium salts, rose bengal and other organic dyes.^12^ Another important photochemical activation mode is based on hydrogen atom transfer (HAT) processes.^13^ In this mode of activation, an excited photocatalyst promotes the generation of a carbon-centered radical upon homolytic cleavage of a C–H bond from an appropriate substrate (typically benzylic, allylic or α-heteroatom abstractable hydrogens). Finally, proton coupled electron transfer (PECT) is another alternative activation mode for the generation of carbon-centered radicals.^14^ This last strategy relies on a concerted 1e^−^/1H^+^ transfer process from an excited photocatalyst, which transiently forms the desired radical intermediates, as it occurs in some cases when ketones are employed as photosubstrates.

Despite the great utility offered by these activation modes, an ideal scenario in terms of sustainability and operational simplicity would be that only the reaction coupling partners and light came into play for the formation of the desired reaction products (Scheme 1b). In the context of photochemical reactions, irradiation with high energy UV light (*λ* < 350 nm) presents a limited applicability, due not only to undesired bond-breaking events promoted by the highly energetic light that normally leads to the formation of undesired by-products, but also related to the toxicity and laboratory security issues associated with the employment of such powerful radiation.^15^ Thus, photochemical approaches based on the more sustainable, safer, and easier to generate visible light as the activation source have caught the attention of organic chemists in the last few years.^16^

An emerging strategy for the generation of carbon-centered radicals combines photochemistry with supramolecular interactions.^17^ The formation of aggregates through non-covalent weak interactions (such as hydrogen bonding interactions or π-effects) represents a smart strategy to modulate reactivity and stereoselectivity in radical photochemical reactions within the molecular assembly. Specifically, the formation of electron donor–acceptor (EDA) complexes stands as a very elegant and successful approach for the generation of carbon-centered radicals.^18^ The key to the success of these light-driven reactions is simple. Typically, when an EDA complex is assembled, a new band in the UV-vis spectrum is observed when compared to the individual species that form the complex. This new charge-transfer (CT) band is due to a photoinduced single electron transfer from the electron donor species to the electron acceptor counterpart (Scheme 1(c.1)). Indeed, when an EDA complex is irradiated at wavelengths within the CT band, a radical ion pair may be formed, which can directly recombine or engage in different processes (such as cyclizations or interception by different radical-trapping agents). Overall, this strategy grants access to carbon-centered radicals under very mild reaction conditions.

A class of EDA complexes that has significantly contributed to the growth of photosynthetic chemistry in recent years are those built upon non-covalent halogen-bond (HB) formation.^19^ The creation of a halogen-bonding complex is due to some specific kind of supramolecular interaction known as σ-hole interaction.^20^ Typically, electron-acceptor molecules that contain a covalently bonded halogen atom present a region of positive electrostatic potential along the extension of the covalent bond, the so-called σ-hole. An attractive non-covalent interaction can potentially occur between an acceptor molecule I and a nucleophile or electron donor species II (Scheme 1(c.2)). Specifically, this interaction consists of a partial n → σ* charge-transfer from a non-bonding orbital of the electron donor (HB acceptor) to an antibonding orbital (σ*) of the electron acceptor (HB donor).^20*e*^ The strength of this interaction has been studied both theoretically and experimentally and was found to be proportional to the amount of n → σ* charge-transfer.^20*c*^ An important feature of the supramolecular interaction between these two partners is its high directionality to form a halogen-bonding complex III,^21^ typically displaying R_1_–X–D dihedral angles between 160° and 180°. As will be described throughout this review, conventional characterization methods for the identification of halogen-bonding complexes include UV-vis, NMR, IR, ITC and ESR.^19*c*,*e*,*f*^ An additional feature of the halogen bond is the flexibility in the employment of different skeletons and halogen atoms (mainly Br and I), which can lead to different binding properties and solubility profiles of the HB complexes. From the photochemical point of view, upon light-driven excitation of the complex, a SET from the donor to the acceptor partners may occur promoting the fragmentation with the loss of the halide anion and generating two different open-shell radical species, IV and V. All of the unique characteristics described above provide chemists with the opportunity to explore novel transformations based on the activation of the halogen bond. Indeed, this photochemical activation mode has been exploited in the last few years for the generation of carbon-centered radicals from organic halides, which can then engage in a wide variety of novel chemical transformations. In this review we intend to give an overview of the most representative examples featuring the photochemical activation of carbon–halogen bonds through EDA and specifically HB complexes for the generation of carbon-centered radicals and their synthetic utility.

## 2 Photochemical halogen-bond activation mode for the generation of carbon-centered radicals

In order to provide the general reader with a better understanding of the different reactivities that emerge from photochemical halogen-bonding assisted reactions, a division in sections depending on the type of radical coming from the halogen-bond donor (alkylic, arylic or alkenylic) has been established.

### 2.1. General mechanisms

**Fig. 1:**
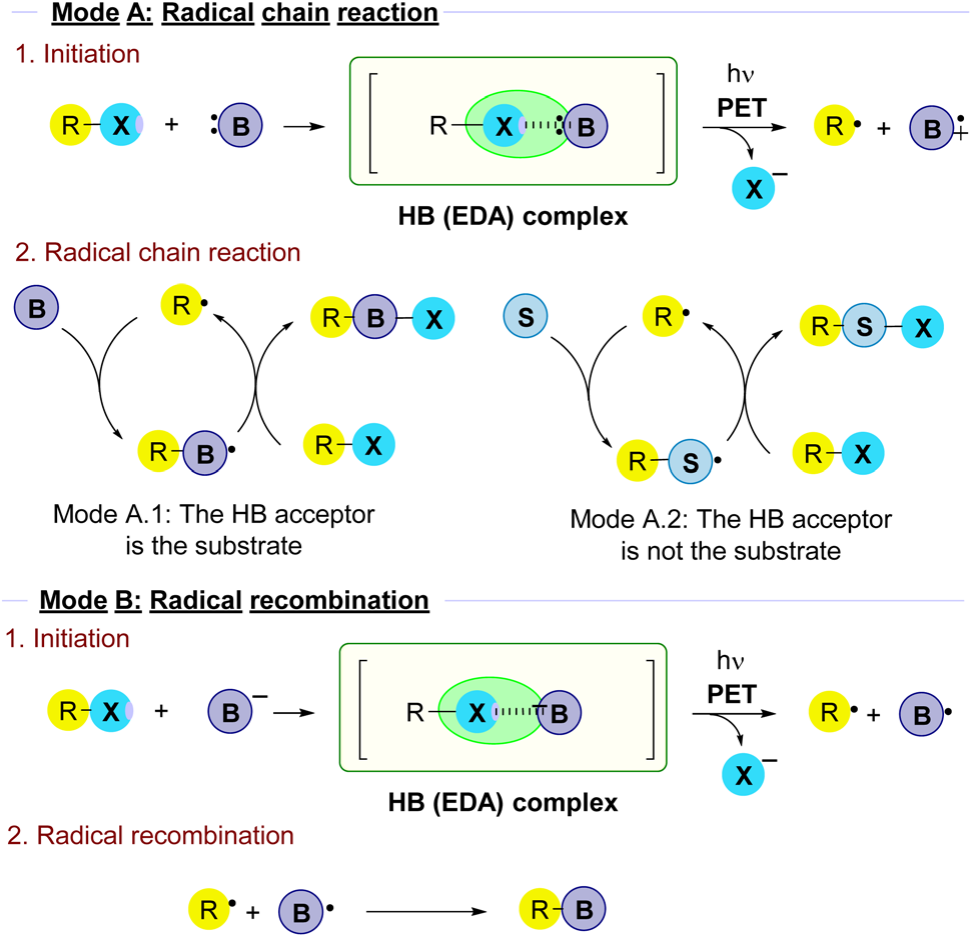
Classification of reactivity modes in radical reactions triggered by the photochemical fragmentation of halogen-bond complexes that will be followed in this review.

There are a variety of mechanisms for the radical reactions which are triggered by the photochemical fragmentation of a halogen bond. In order to organize the results in this review attending to mechanism similarities on the overall transformations, a main classification has been made (Fig. 1).

#### 2.1.1 Mode A: radical chain reaction

The processes occur through a radical chain reaction. The light-promoted fragmentation of the HB complex is the initiation step in the radical transformation and is responsible for the generation of the alkyl radical, that then enters the propagation steps. The determination of the existence of a radical chain reaction is typically carried out by the measurement of the quantum yield or light on/off experiments. Quantum yields *φ* > 1 are an indication of the existence of a radical chain reaction. Among the processes that involve a radical chain reaction two main types of transformations can be distinguished.

##### 2.1.1.1 Mode A.1: the HB acceptor is the substrate

In these reactions a HB-complex is formed between the alkyl halide and a halogen bond acceptor, typically a Lewis base B. Upon fragmentation, the alkyl radical reacts with another molecule of B in the radical chain process to ultimately produce the coupling product.

##### 2.1.1.2 Mode A.2: the HB acceptor is an external promoter

In this type of transformation, upon fragmentation the alkyl radical reacts with a different species S in the radical chain reaction. Thus, the role of the Lewis base B as a HB acceptor is just to act as an external promoter in the initiation step.

#### 2.1.2 Mode B: radical recombination

Upon fragmentation, the open shell species generated directly recombine to form a coupling product. In this case, no radical chain reaction occurs. Quantum yields *φ* ≪ 1 are an indication of the absence of a radical chain reaction.

Along the review, in every section the different examples will be grouped attending to this general classification.

### 2.2. Common HB donors and acceptors

Apart from the mechanistic considerations, in each section the selected examples covered in this review have been organized attending to the nature of the halogen bond donors and acceptors.

**Fig. 2:**
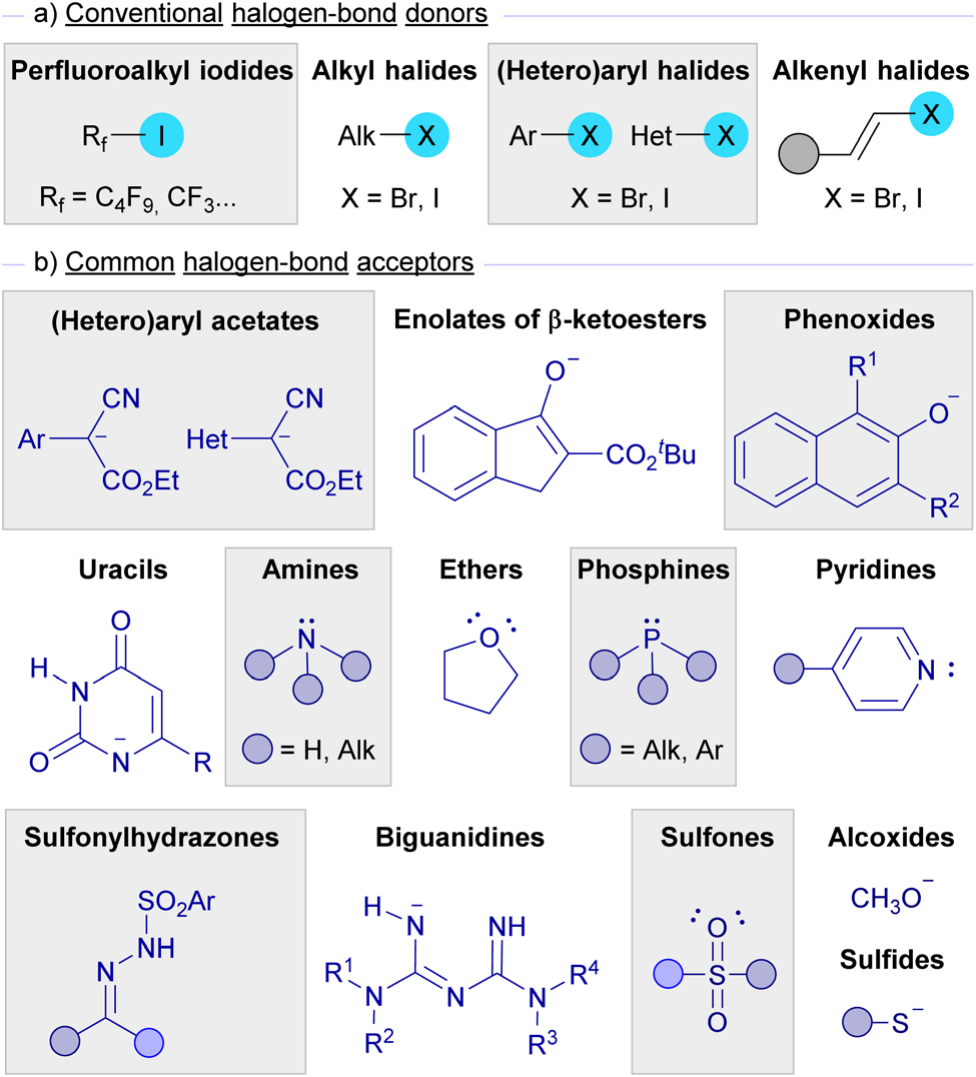
Summary of the most common halogen bond donors and acceptors employed in photochemical HB assisted reactions.

As depicted in Scheme 1, halogen bond donors are alkyl, (hetero)aryl and alkenyl halides, where a subclass of alkyl iodides, perfluoroalkyl iodides, has been extensively used in this kind of photochemical reaction. On the other hand, nucleophilic species such as enolates, sulfides, alcoxides, and amines are the most widely used halogen-bond acceptors, while other nucleophilic partners like sulfonylhydrazones, sulfones, phosphines, biguanidines, and uracils have also been found to be successful for the formation of halogen-bond complexes. Fig. 2 presents an overview of the halogen-bond donors and acceptors that have been discussed in this review.

It must be noted that there are several examples of photochemical activation of C-halogen bonds which have been identified by the authors to proceed *via* EDA complexes but not specifically HB-complexes. It is not our intention to go against the original interpretation of the authors and therefore, the supramolecular complexes will be treated as EDA or HB depending on how it is reported in the original publications. Additionally, those reactions in which the EDA complexes are clearly identified as non-HB-complexes will not be covered in this review.

### 2.3. Reactions initiated by the generation of C sp^3^-centered radicals

#### 2.3.1 Perfluoroalkyl carbon-centered radicals

The most extensive studies on the generation of radical species in photochemical halogen-bonding initiated reactions have been reported on the creation or alkyl radicals. Among them, the most broadly used halogen-bond donors in these of transformations are perfluoroalkyl iodides 3 (R_f_–I, *R*_f_ = C_*n*_F_*n*+1_), which act as the carbon radical precursor.^22^ Their strongly electron-deficient nature makes them excellent electron-acceptors for their participation in the formation of HB bonding complexes with Lewis bases. As discussed above, the different examples have been classified based on the type of mechanism.

##### 2.3.1.1 Mode A.1: radical chain reactions with the HB acceptor as the substrate

**Scheme 2:**
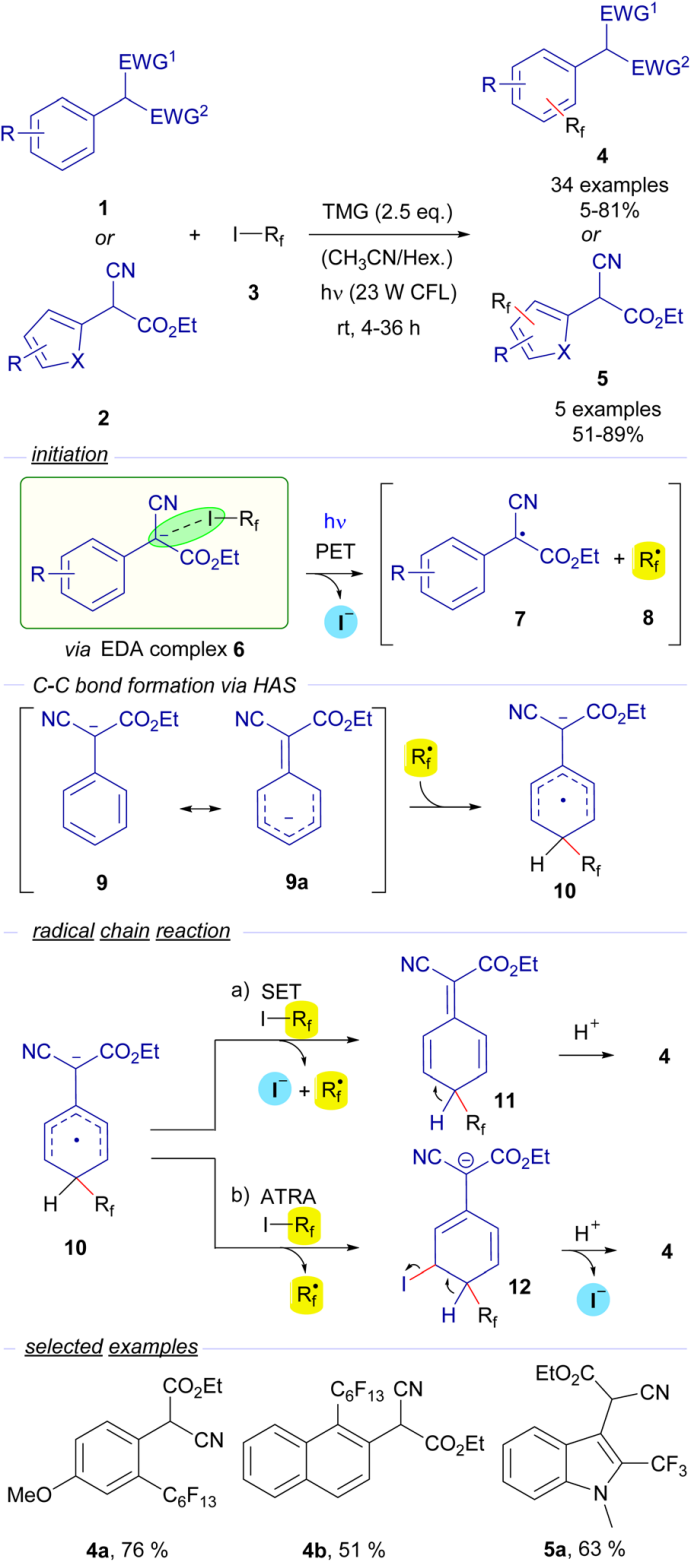
Direct photocatalyst-free aromatic perfluoroalkylation and trifluoromethylation of α-cyano (hetero)arylacetates 1 and 2. TMG: 1,1,3,3-tetramethylguanidine, CFL: Compact Fluorescent Lamp (400–700 nm), HAS: Homolytic Aromatic Substitution, and SET: Single Electron Transfer.

The first example of the employment of perfluoroalkyl iodides in catalyst-free photochemical transformations was reported by Melchiorre and co-workers. In a seminal publication in 2014, they described a novel visible-light-induced direct perfluoroalkylation and trifluoromethylation of α-cyanoarylacetates 1 and their corresponding heterocyclic analogues 2 (Scheme 2).^23^ In these reactions the perfluoroalkylation occurs on the aromatic ring of the arylacetates. An EDA complex 6 is believed to be formed between the perfluoroalkyl iodides 3 and the enolate of the cyanoaryl acetates 1. It must be noted that the nature of the supramolecular interaction in the EDA complex 6 has not been described as a halogen-bond complex in this seminal example. Nevertheless, considering this reaction as an early example of photochemical activation of a carbon–iodide bond *via* EDA complex formation, we found it pertinent to include this reaction in this review. The examination of the UV-vis absorption profiles revealed a red-shifted charge-transfer band when a mixture of 1, 3 and TMG was prepared in MeCN, which is most likely due to the formation of the EDA complex 6. Photoexcitation with visible light fragmentates the adduct and generates the radical species 7 and 8. Preliminary mechanistic studies involving a quantum yield determination (*φ* = 3.8) revealed that radical chain propagation was occurring in this transformation. To explain this observation, the authors propose a HAS (homolytic aromatic substitution) mechanism.

The perfluoroalkyl radical 8 is postulated to react with enolate 9 (which has a mesomeric form 9a), forming a radical anion intermediate 10. From this point, two different reaction pathways can lead to the formation of the final products 4 by reaction with the perfluoroalkyl iodide 3 through the intermediates 11 and 12*via* SET or ATRA events respectively, releasing another unit of the perfluoroalkyl radical 8, that is incorporated in the first step of the chain reaction. From a synthetic point of view, the transformation consists of the *o*- and/or *p*-perfluoroalkylation of the α-arylcyanoacetates through an operationally simple methodology. Conceptually, this work introduced for the first time the employment of perfluoroalkyl iodides as sources or perfluoroalkyl radicals through the formation of EDA complexes, a strategy that has been thoroughly applied since then. Moreover, it also validated the feasibility of the employment of enolates as suitable partners in HB-complex based photochemical processes.

A year later, the Melchiorre group reported another catalyst-free perfluoroalkylation reaction employing the combination of perfluoroalkyl iodides and phenols under basic conditions.^24^ However, a different mechanism has been proposed for the photochemically driven reaction that did not involve the formation of EDA-complexes and therefore will not be discussed herein.

**Scheme 3:**
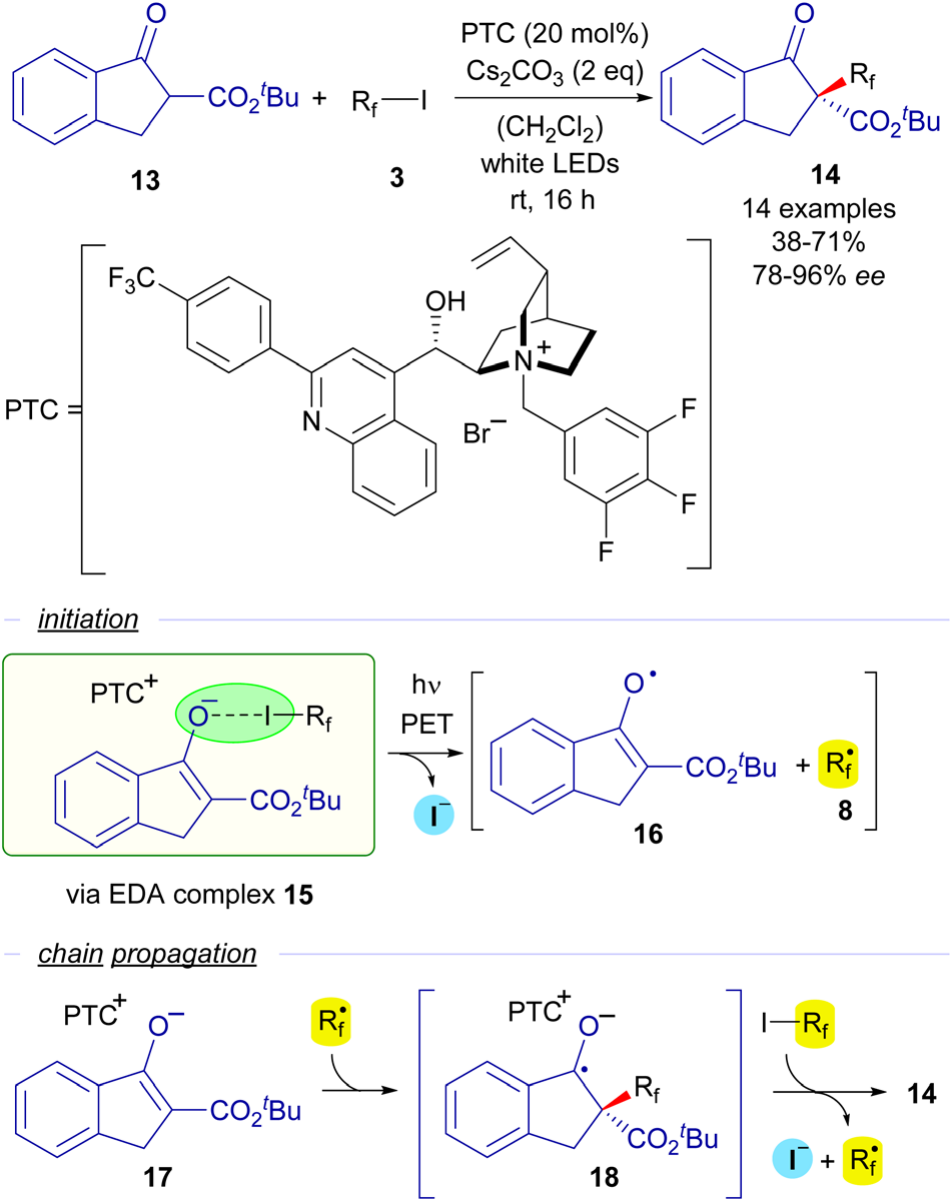
Enantioselective perfluoroalkylation of β-ketoesters 13.

The first enantioselective approach to a photocatalyst-free halogen-bonding assisted transformation was developed also by the group of Melchiorre in 2015 (Scheme 3).^25^ The reaction consists of an enantioselective perfluoroalkylation of cyclic β-ketoesters mediated by a chiral cinchona-derived phase transfer catalyst (PTC). When mixing in a dichloromethane solution a β-ketoester 13, a perfluoroalkyl iodide, Cs_2_CO_3_ as the base and the PTC, a yellow-coloured solution was obtained, which was indicative of the formation of a HB complex 15 in the media. This fact was confirmed by a UV-vis analysis, where a red-shifted CT band was observed for this mixture of reactants. Irradiation with white LEDS enabled the formation of perfluoroalkylated ketoesters 14 with very good yields and excellent levels of enantioselectivity. The mechanism of this asymmetric perfluoralkylation reaction would consist of an initiation step in which the halogen-bonding adduct 15 is photoexcited and two different radical species 8 and 16 are formed. A radical chain propagation is proposed by the authors to occur. First a radical addition of the perfluoroalkyl radical onto the starting enolate 17 (which is presumably bounded to the chiral cinchona PCT through π–π stacking interactions) takes place to form a new radical anion intermediate 18. Next, 18 reacts with another molecule of the starting perfluoroalkyl iodide *via* SET, initiating the radical propagation and enabling the formation of the perfluoroalkylated ketoester 14 with high enantioselectivity. This reaction sets a precedent as the first enantioselective transformation initiated by visible light promoted HB activation.

Two years later, in 2017, the groups of Li and Cheng tried to rationalize the stereoselectivity observed in this Melchiorre's reaction through an in-depth computational study.^26^ Their calculations suggest that multiple hydrogen-bonding interactions between the enolate 17 and the PTC, rather than π–π stacking, are responsible for the origin of the enantioselectivity in their transformations.

**Scheme 4:**
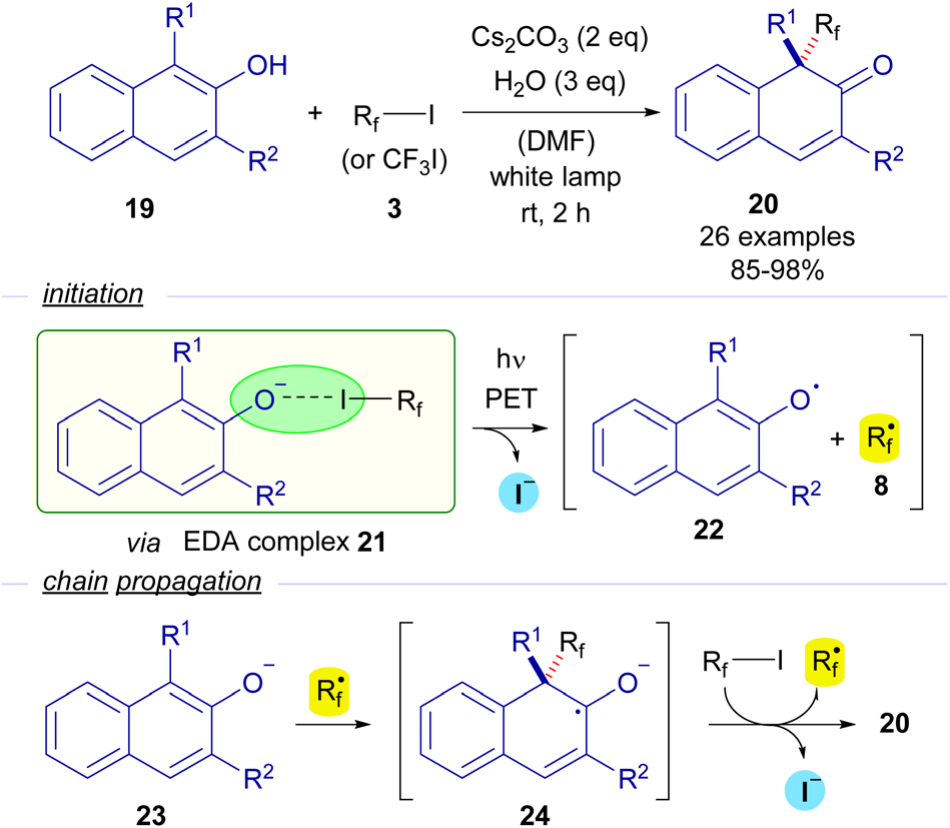
Visible light promoted fluoroalkylation of β-naphthols 19.

In 2018, Wang and Xu reported the first photochemical dearomatizative fluoroalkylation of β-naphthols 19 carried out without the need for any transition metal or external photosensitizer (Scheme 4).^27^ The reaction is initiated by the formation of the EDA complex 21, whose existence was justified thanks to UV-vis analysis. The photoexcitation of this adduct with white light enabled the formation of naphthoxide radical 22 and perfluoroalkyl radical 8. A radical chain propagation was proposed based on quantum yield measurements (*φ* = 14). A complete inhibition of the reaction by the addition of TEMPO was observed, which is indicative of the participation of radical species in the transformation. Taking all of these into consideration, the authors proposed that starting from β-naphthoxide 23, a radical addition of 8 would create a new ketyl radical anion 24. As explained before, this intermediate is a strong reductant and is able to reduce another molecule of the perfluoroalkyl iodide, starting the radical propagation and forging the final dearomatized product 20.

**Scheme 5:**
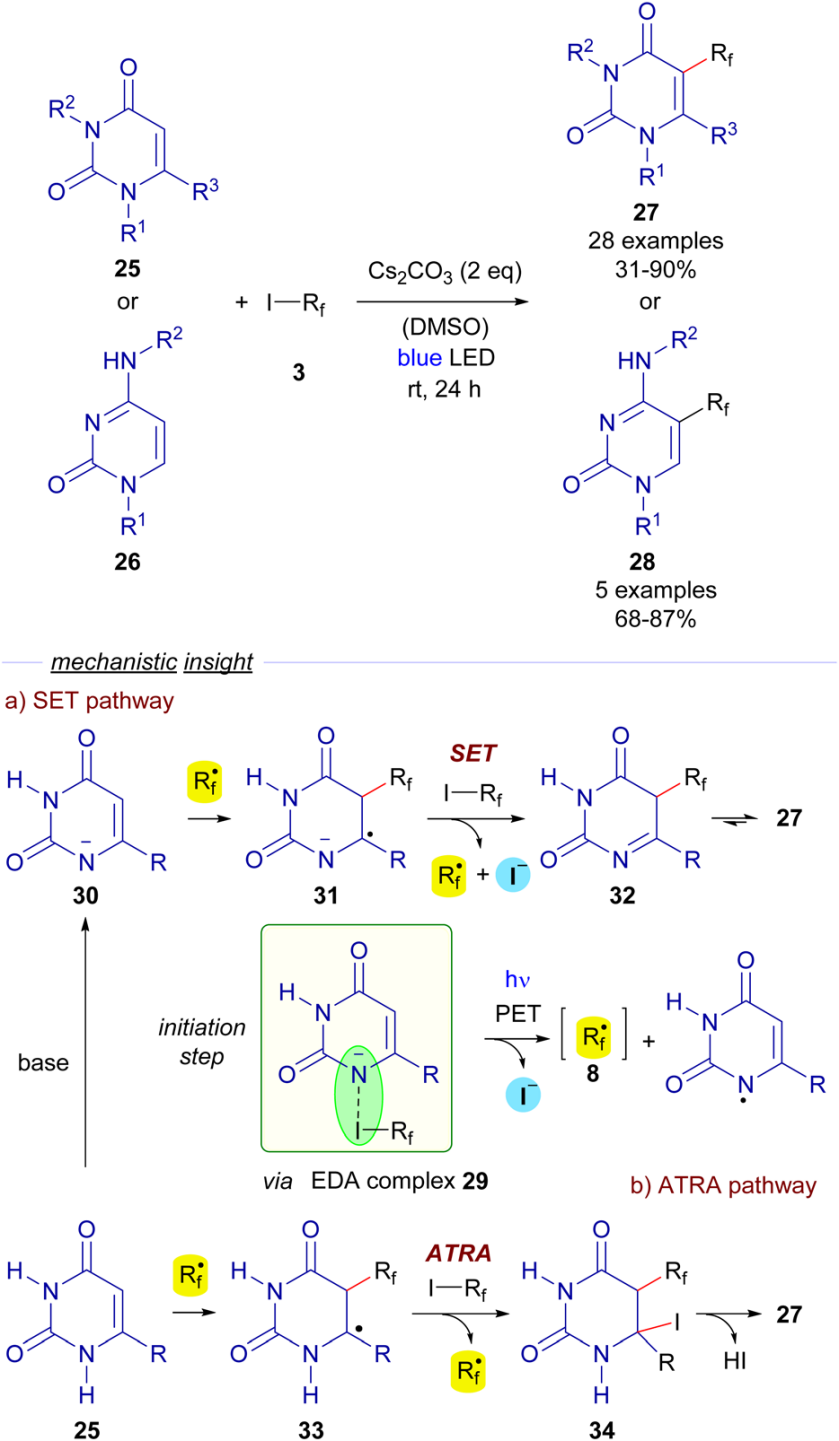
Hydroxylperfluoroalkylation reaction of uracyls and cytosines.

Also in 2018, Li, Zhang and He reported a trifluoromethylation and perfluoroalkylation reaction of uracils 25 and cytosines 26 based on a photochemical activation of EDA complexes (Scheme 5).^28^ In particular, the formation of an EDA complex 29 was detected by UV-vis analysis of a solution of a perfluoroalkyl iodide 3 and the deprotonated uracil 30.

Photoexcitation with blue light of the EDA complex would provide perfluoroalkyl radical 8. After this event, the authors propose two different pathways for the formation of the final polifluoroalkylated uracils 27. The SET pathway (Scheme 5) would involve the radical addition of 8 to the deprotonated uracil 30 to form a new radical anion species 31, which engages in a SET event with another equivalent of 3, starting a radical propagation by regenerating the perfluoroalkyl radical 8 and the imine 32, which tautomerizes to form the perfluoroalkylated uracyl 27. Alternatively, the ATRA pathway would consist of a radical addition of 8 to the uracil 25 to form the new radical intermediate 33, which abstracts an iodine atom from 3 to generate the iodinated compound 34 and starts a radical chain propagation by regenerating of 8. The elimination of HI would eventually produce the functionalized uracils 27. Identical mechanisms can be proposed for the conversion of cytosines 26 into 28. Overall, this transformation represents a metal-free, operationally simple approach towards the synthesis of novel biologically active cytosines and uracils under mild conditions.

##### 2.3.1.2 Mode A.2: radical chain reactions with an external promoter HB acceptor

In the seminal examples by the Melchiorre group discussed above the photochemical excitation of the HB-bond (EDA) complex is responsible for the initiation of the radical chain reaction, and the enolate that forms the complex with the perfluoroalkyl iodide is also the substrate that reacts with the perfluoroalkyl radical in the propagation step.

**Fig. 3:**
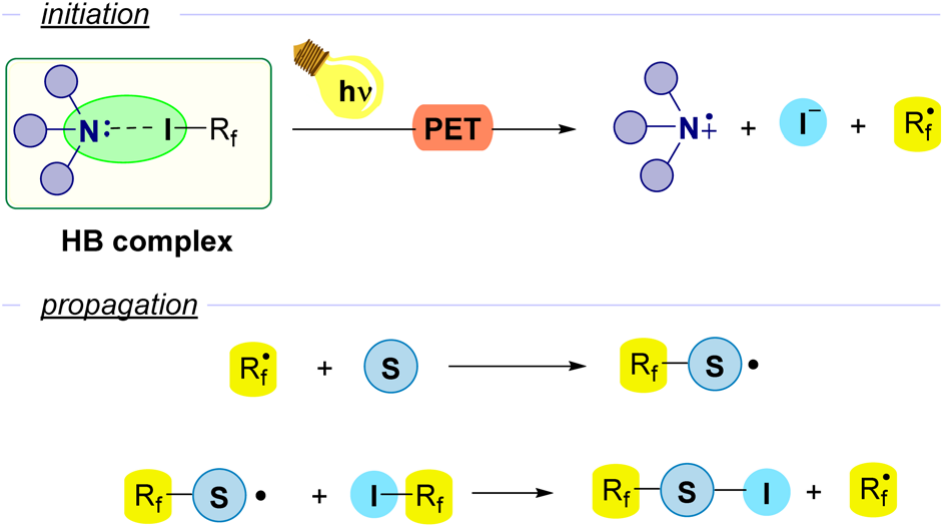
General scheme of photochemically halogen-bond-promoted radical chain reactions with perfluoroalkyl iodides using an amine as an external promoter.

However, another approach that has been extensively employed consists in the use of a Lewis base as an external promoter, that participates in the initiation step as a HB-acceptor but does not participate in the propagation and is not incorporated into the final product. In particular, amines are the most widely used promoters for the generation of R_f_ radicals. Upon photoinduced electron transfer (PET) the perfluoroalkyl radical is formed with release of iodide and the radical cation derived from the amine. Then, in the first step of the propagation sequence the perfluoroalkyl radical reacts with a radical acceptor substrate. The evolution of the newly formed radical through a variety of reactions releases the final product and generates a new unit of the perfluoroalkyl radical R_f_˙. This general scheme is found as the initiation step for a number of radical chain reactions (Fig. 3).

**Scheme 6:**
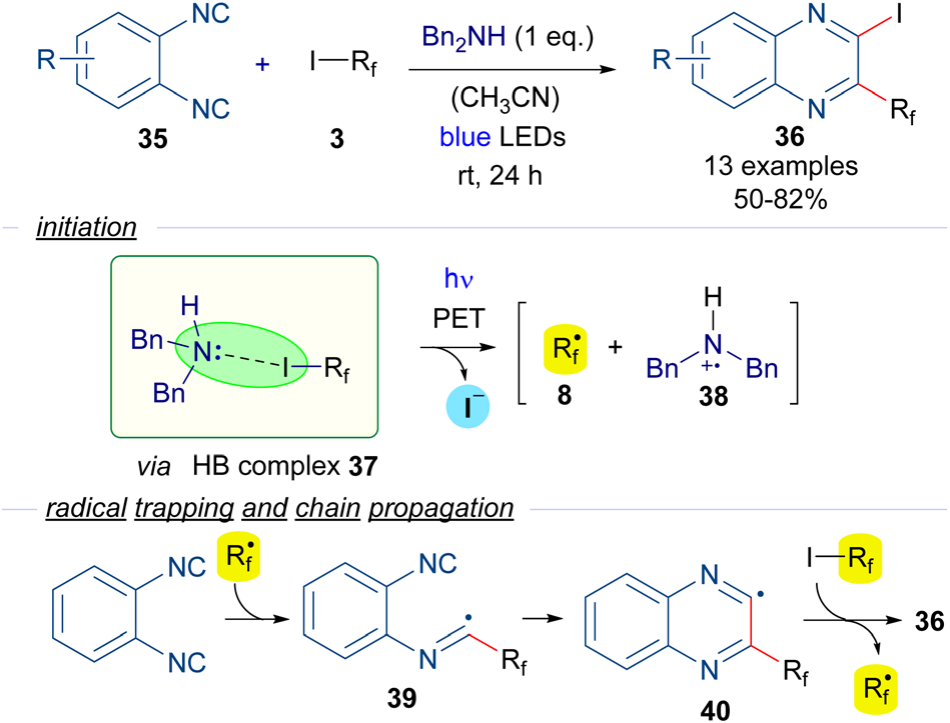
Halogen-bond-promoted double radical isocyanide insertion with perfluoroalkyl iodides 3.

For instance, in 2016 Ma and Yu reported a halogen-bond-assisted double radical isocyanide insertion employing perfluoroalkyl iodides and 1,2-diisocyanobenzenes 35 to get 2-iodo-3-perfluoroalkylated quinoxalines 36 (Scheme 6).^29^ The combination of dibenzylamine as an electron donor with the perfluoroalkyl iodides 3 gives rise to the formation of the HB complex 37, the presence of which was supported by DFT calculations and ^19^F-NMR titration experiments. Irradiation with blue light promotes a PET and fragmentation of the perfluoroalkyl iodide to form the perfluoroalkyl radical 8, which is trapped by one of the isocyanide groups of 25, forming the intermediate 39. A subsequent intramolecular radical addition would form the quinoxalinyl radical 40, which starts the radical chain propagation *via* abstraction of an iodine atom from another unit of 3 to generate perfluoroalkyl radical 8 and the disubstituted quinoxaline 36. The presence of free radicals in this reaction was supported by Electronic Paramagnetic Resonance (EPR) experiments employing 3 and *tert*-butyl-α-phenylnitrone as the spin trapping agent.

**Scheme 7:**
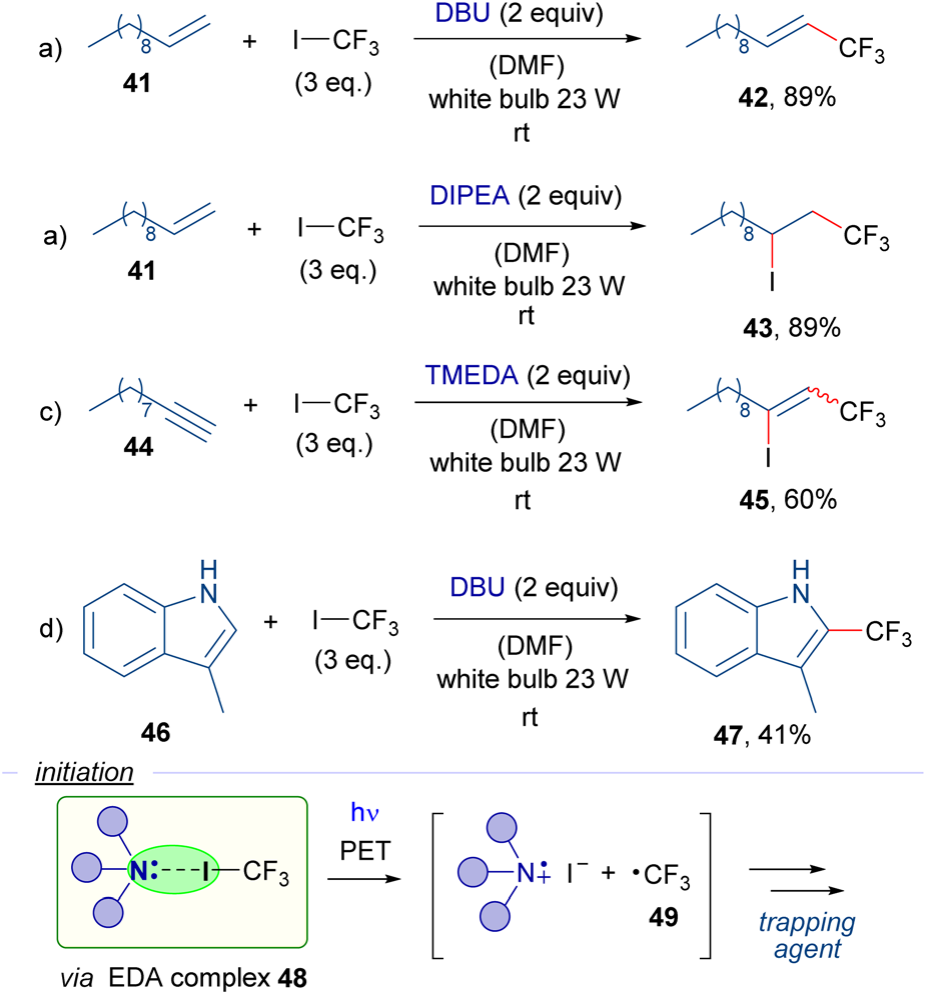
Visible-light-induced catalyst-free trifluoromethylation reactions with white light.

Also in 2016, the research group of Cho reported a series of trifluoromethylation reactions employing trifluoromethyl iodide, organic Lewis bases and different radical trapping agents as the reaction partners (Scheme 7).^30^ It is worth mentioning that most of this work is based on a photosensitization of the CF_3_I molecules by the photocatalyst (Nile red) under exposure to yellow light. Nevertheless, the authors also reported the catalyst-free version of some of their reactions. Halogen-bond complexes 48 are postulated to be formed between CF_3_I and the amines, which upon photoexcitation with white light would generate the trifluoromethyl radical 49 that then is trapped by various radical acceptors such as alkene 41, alkyne 44 and indole 46 to produce the different trifluoromethylated compounds 42, 43, 45 and 47, respectively.

**Scheme 8:**
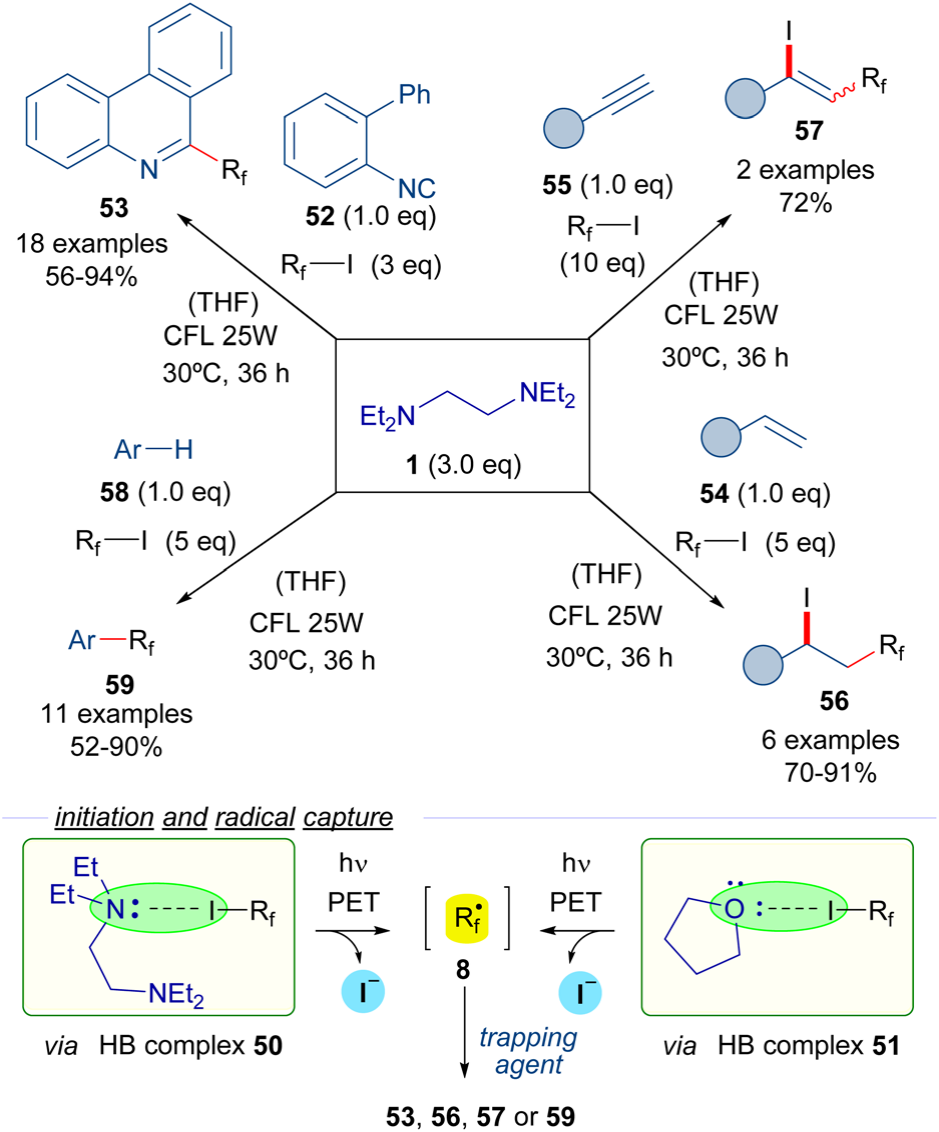
Perfluoroalkylation reactions of unsaturated systems initiated by irradiation of the halogen-bonding complexes 50. TEEDA: *N*,*N*,*N*′,*N*′-tetraethylethylenediamine.

In 2017, Chen *et al.* reported the use of TEEDA as the halogen bond acceptor in a set of perfluoroalkylation reactions (Scheme 8).^31^ Upon testing a collection of organic bases, TEEDA was proven to be an optimal promoter for the reactions based on perfluoroalkyl radicals. The formation of the halogen-bond complex 50 with the perfluoroalkyl iodides 3 was detected by ^19^F-NMR titration experiments. The photoexcitation of complex 50 with low-intensity irradiation (CFL, UV or even sunlight) provides the perfluoroalkyl radicals 8 that can be trapped by several radical acceptors. Moreover, the authors suggest that an additional HB complex 51 can potentially be formed between the perfluoroalkyl iodides and the THF molecules of solvent, which would also lead to the generation of the perfluoroalkyl radicals. These perfluorinated radical intermediates were initially trapped with 2-isocyanobiphenyls 52 to furnish different phenantridines 53. Next, various alkenes 54 and alkynes 55 were selected as radical trapping agents to achieve the iodo perfluoroalkylation of the unsaturated bonds to form the iodo-perfluoroalkylated alkanes 56 and alkenes 57 respectively. Finally, a C–H perfluoroalkylation reaction of arenes and heteroarenes 58 was developed for the synthesis of perfluoroalkylated arenes 59. This protocol could be also applied to the site-selecting labelling of oligopeptides at a tryptophan residue.

**Scheme 9:**
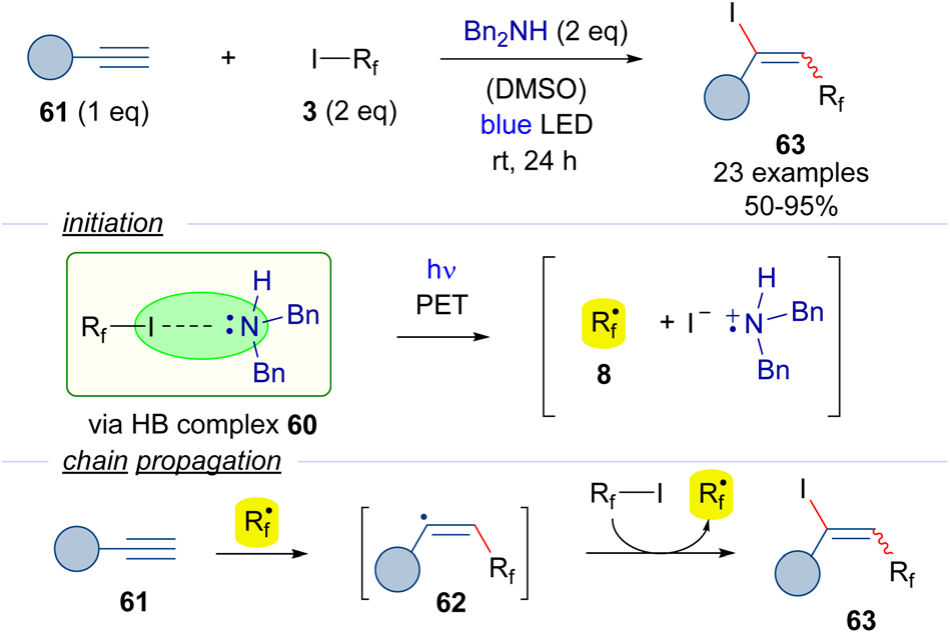
Synthesis of 1,2-difunctionalized alkenes 63 by iodo perfluoroalkylation of alkynes.

Another version of the iodoperfluoroalkylation of alkynes was reported by Yu in 2018 (Scheme 9).^32^ In this work, dibenzylamine was used as the halogen bond acceptor. The photoexcitation of the HB adduct with blue light would enable the formation of the radical intermediate 8. A radical chain propagation event was hypothesized to occur, where an alkenyl-centered radical 62 could be formed by the addition of the perfluoroalkyl radical 8 to the alkyne 61. The transfer of an iodine atom from 3 to 62 would complete the radical propagation generating the iodoalkene 63 and another radical 8. Radical-clock experiments proved the presence of radical intermediates being formed during the transformation.

Interestingly, a flow protocol for the perfluoroalkylation of olefins was reported in 2019 by the group of Kappe through the photochemical activation of EDA complexes arising from the combination of perfluoroalkyl iodides 3 and Et_3_N as the HB acceptor employing blue light irradiation.^33^ As an important application, their protocol allowed for the gram-scale synthesis of pharmaceutical ingredients in the flow.

**Scheme 10:**
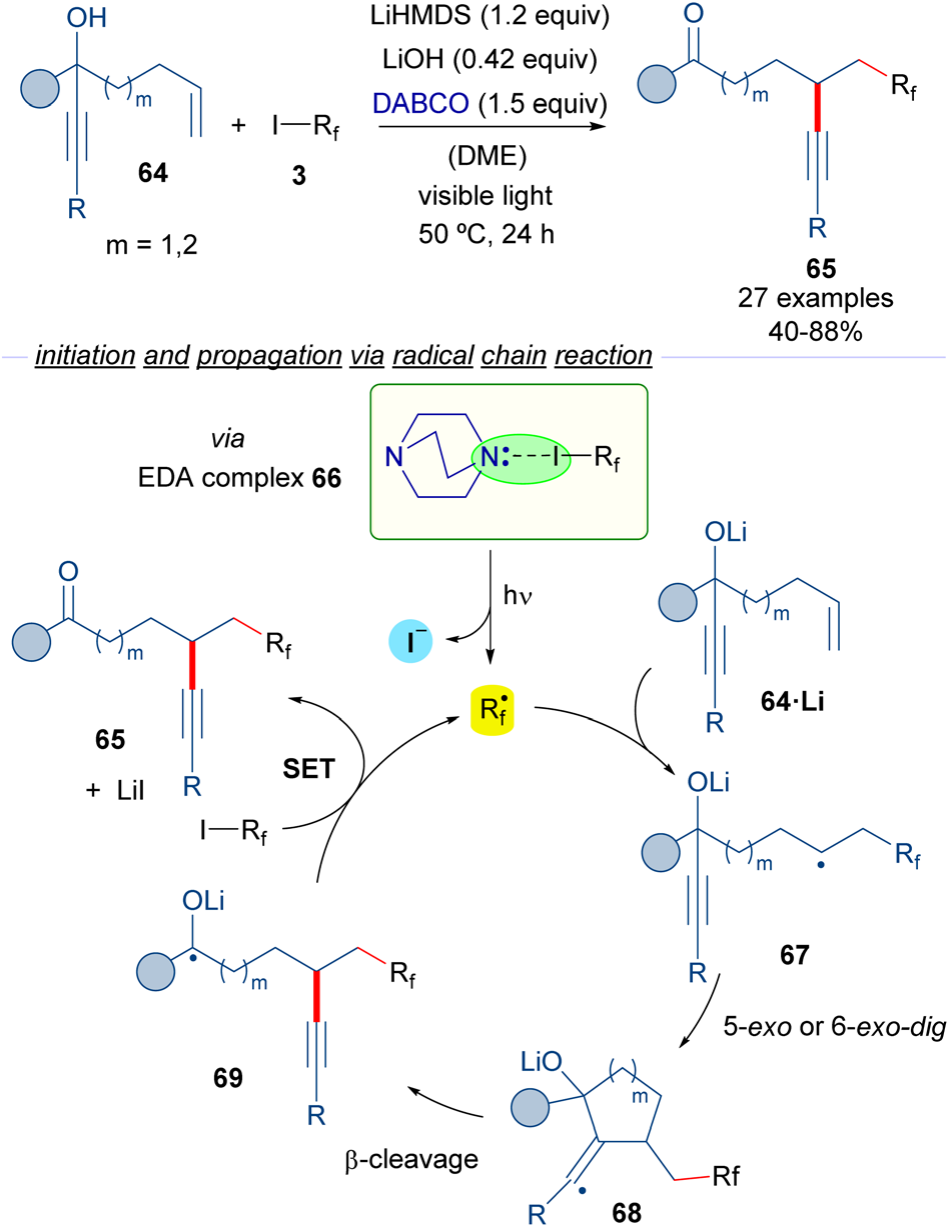
Transition metal-free radical α-perfluoroalkylation with concomitant β-alkynylation of unactivated alkenes. DABCO: 1,4-diazabicyclo[2.2.2]octane.

The group of Studer reported in 2017 reported a photochemical transformation for the vicinal difunctionalization of alkenes involving a perfluoroalkylation reaction (Scheme 10).^34^

In this transformation, different perfluoroalkyl iodides 3, an amine and a radical trapping agent are employed. The reaction design is based on the employment of the propargyl alcohols 64 featuring a tethered double bond at an appropriate distance as radical acceptors. The photoexcitation of the HB complex 66 formed between DABCO and the perfluoalkyl iodide generates the corresponding perfluoroalkyl radical, which adds to the double bond of 64 to form the radical intermediate 67. A subsequent 5- or 6-*exo-dig* radical cyclization followed by β-C–C bond cleavage would form the radical 69, which is oxidized by a molecule of 3, to form the reaction product 65, and another unit of the perfluoroalkyl radical that continues the radical chain process. All in all, a novel metal-free α-perfluoroalkylation β-alkynylation of unactivated olefins *via* 1,4- or 1,5-alkynyl migration was possible taking advantage of the halogen-bonding-triggered formation of perfluoroalkyl radical species that start the radical cyclization process.

**Scheme 11:**
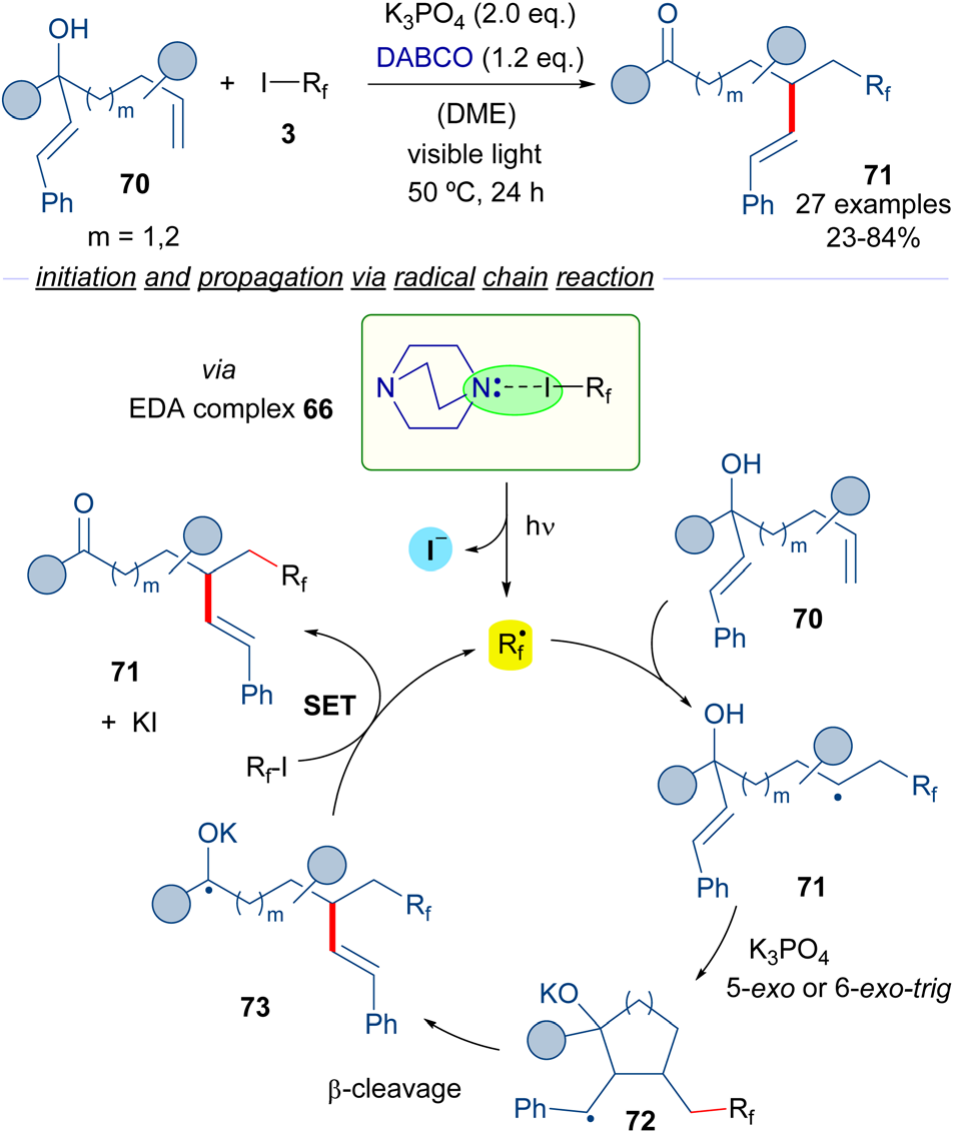
Alkene 1,2-difunctionalization reaction by radical migration.

Subsequently, the same group reported a similar transformation based on the employment of hepta-1,6-dien-3-ols and octa-1,7-dien-3-ols 70 as radical acceptors (Scheme 11)^35^ in a process that consists of the vicinal perfluoroalkylation/alkenylation of an unactivated double bond to form ketones 71. Interestingly, in the reaction an unprecedented 1,4- or 1,5-alkenyl migration takes place. Mechanistically, the reaction is again started by a photochemical activation of the complex 66, the fragmentation of which leads to the formation of the perfluoroalkyl radical *R*_f_·. The same cascade as the previously described transformation occurs, which involves a radical addition to 70 to form a new radical intermediate 71, which engages in a 5-*exo* or 6-*exo-trig* cyclization followed by β-cleavage to provide the radical anion intermediate 73. This intermediate reacts with another molecule of 3, restarting the catalytic cycle and forging the desired final products 71.

**Scheme 12:**
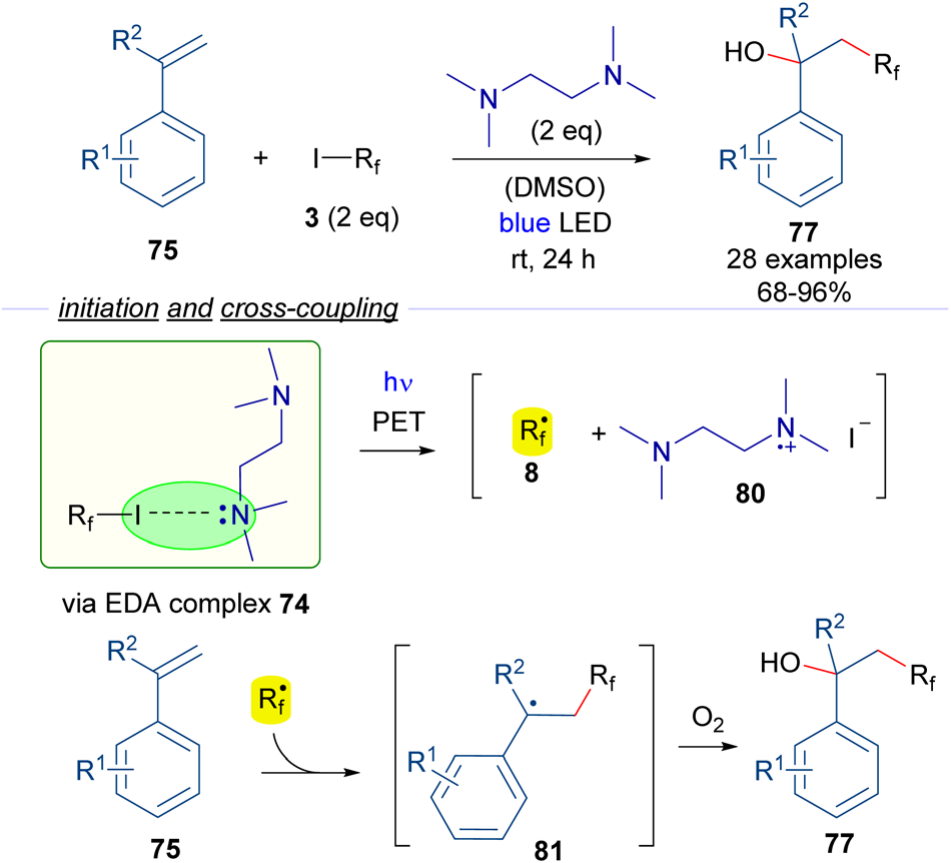
Hydroxy perfluoroalkylation reaction of alkenes promoted by TMEDA.

In another study on halogen-bonding assisted photoreactions based on HB complexes between perfluoroalkyl iodides and tertiary amines, Huang and Chen reported the hydroxy perfluoroalkylation of alkenes (Scheme 12).^36^ An EDA complex 74 was proposed to be formed between the starting perfluoroalkyl iodide and TMEDA, due to successful ^19^F-NMR titration experiments. Photoexcitation with blue visible light of the halogen-bonding complex 74 generates the active radical species 8. Radical addition to the styrenes 75 forms the benzyl radical intermediate 81 which reacts with molecular oxygen to form the oxidized products 77. In this case, the quantum yield determination (*φ* = 0.23) excluded the possibility of radical chain propagation processes during the reaction. From a synthetic perspective, the reaction consists of the 1,2-addition of a hydroxy and a perfluoroalkyl group to the double bond of the alkene under catalyst-free conditions.

**Scheme 13:**
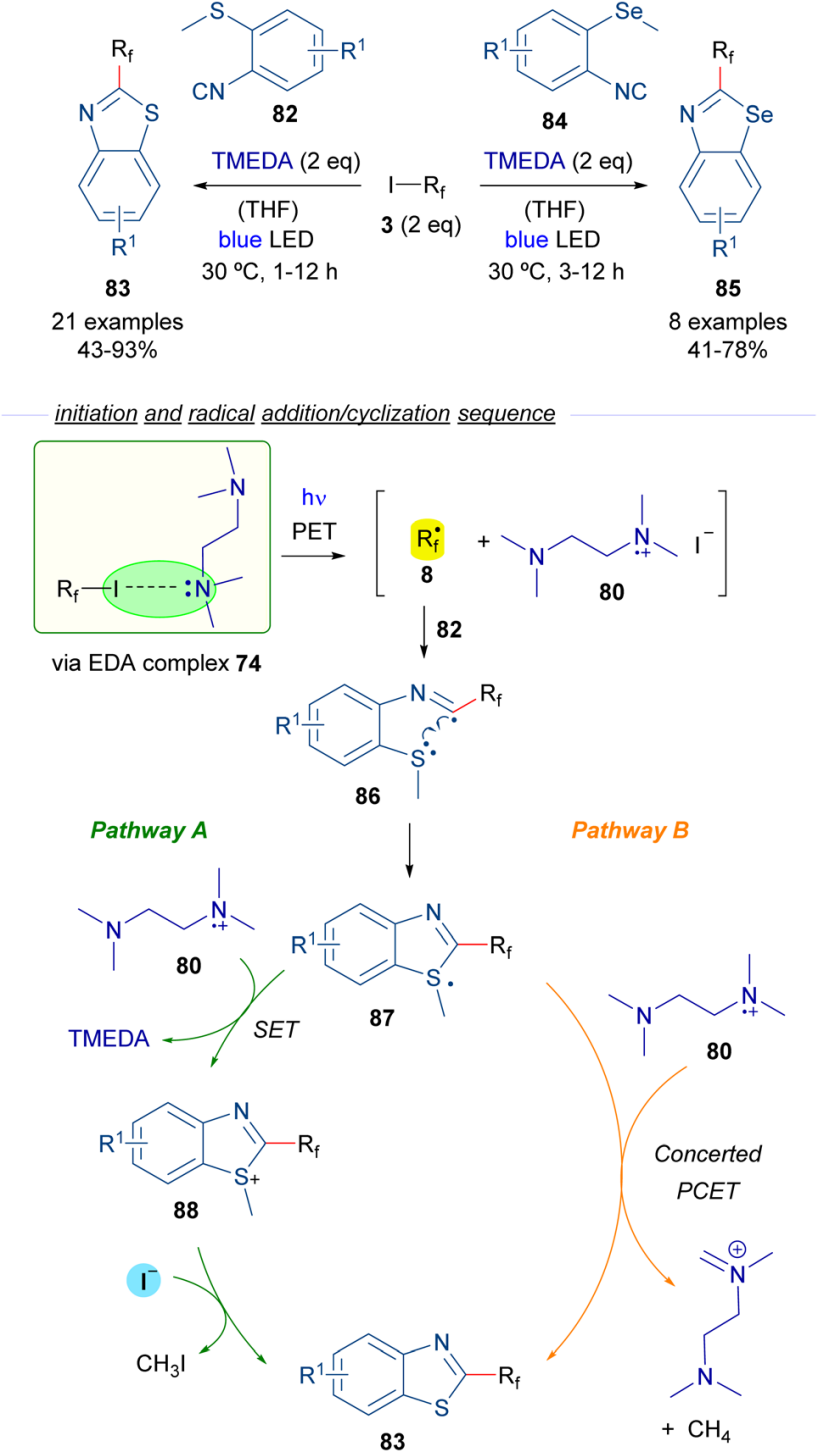
Photochemical HB assisted synthesis of 2-perfluoroalkylbenzothiazoles/benzoselenazoles.

In 2019, Yu and Chen reported a perfluoroalkylation/cyclization strategy for the preparation of 2-perfluoroalkylbenzothiazoles 83 and benzoselenazoles 85 based on the photoinduced generation of perfluoroalkyl radicals (Scheme 13).^37^ Again TMEDA was chosen as the HB donor for the generation of radicals 8 (like in Scheme 12). In the presence of *o*-methylthioisocyanide 82, the addition of radical 8 to the isocyanide generates new radical intermediate 86, which experiences a fast intramolecular cyclization to construct the benzothiazole ring of 87, which contains a sulfur-centered radical. At this point, the reaction is likely to proceed *via* two different pathways. In pathway A, the radical intermediate 87 is oxidized by the radical cation 80 to regenerate TMEDA and produce a sulfonium cation 88. The final benzothiazole 83 is produced through a nucleophilic attack by I^−^ on the methyl carbon within cationic intermediate 88. This reaction also generates MeI as a byproduct, which then undergoes a nucleophilic attack by TMEDA, leading to the formation of the corresponding quaternary ammonium salt. Pathway B contemplates a PCET event from 80 to 87, generating in a single step the final product 83, methane and the cationic imine salt of TMEDA as byproducts. Similar reactions could be performed starting from the analogous selenium isocyanide 84. EPR experiments were run to support the radical mechanism proposed. Additionally, radical scavengers such as TEMPO or BHT were found to completely suppress the reaction, giving additional support to the radical pathway. In this regard, the quantum yield of the transformation was also measured (*φ* = 0.2), excluding the possibility of radical chain propagation processes.

**Scheme 14:**
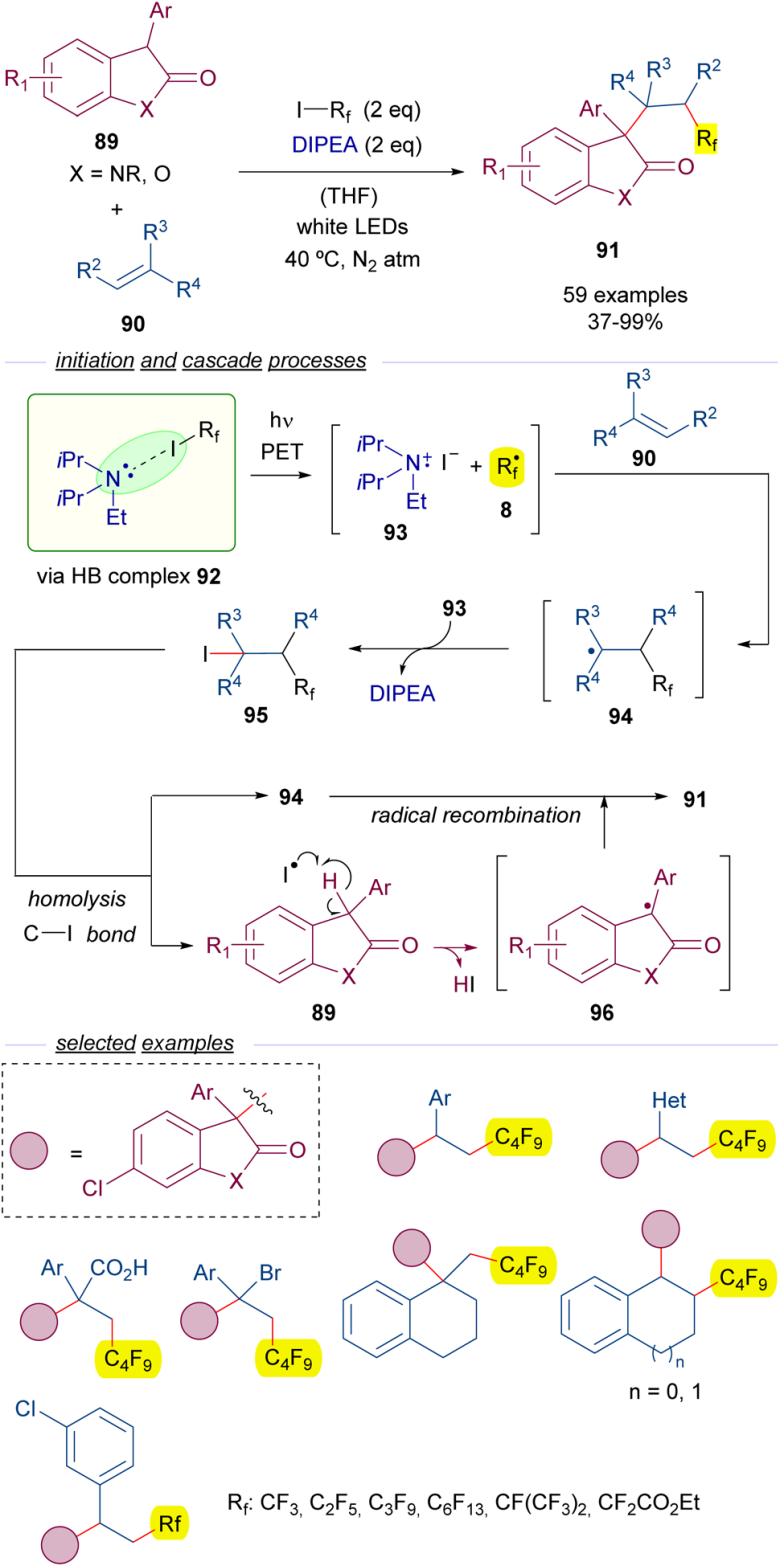
Photochemical catalyst-free three-component synthesis of polyfluorinated oxindoles 91.

Another example of perfluoroalkylation reactions initiated by the formation of a HB complex with perfluoroalkyl iodides and amines is the work reported by Zheng and co-workers in 2021 on the alkylation of oxindoles 89 with olefins 90 (Scheme 14).^38^ Importantly, this reaction represents a novel access to substituted oxindoles 91 with an all-carbon quaternary center. A quite complex mechanism is proposed for this transformation. This time, a light-driven fragmentation of HB complex 92 formed by the assembly of DIPEA and the perfluoroalkyl iodides 3 is responsible for the generation of the two different reactive radical intermediates 8 and 93. Subsequently, the perfluoroalkyl radical adds to the olefin 90 to form the allyl radical intermediate 94, which is oxidized by 93 to form a cationic intermediate that recombines with the iodide anion to give the iodide 95, which is believed to be the key intermediate of this transformation.

A photoinduced homolytic cleavage of the C–I bond of 95 regenerates the radical species 94 and creates an iodide radical, which behaves as a HAT reagent and abstracts the benzylic hydrogen of the oxindole 89, generating a new radical intermediate 96. This step was studied through DFT calculations and a series of control experiments, which supported the critical role of the iodide radical as the hydrogen abstractor. Eventually, the recombination of the radical species 94 and 96 gives rise to the polifluoroalkylated oxindoles 91. Radical clock experiments and successful trapping of the benzyl radical 96 by TEMPO gave evidence to the radical pathway proposed for this reaction. In this three component reaction the perfluoroalkyl group and the oxindole are added at the 1,2 positions of an alkene. A remarkable structural variety of olefins are compatible with the transformation allowing for the synthesis of a set of diversely substituted oxindoles with a perfluoroalkyl substituent. Among the synthetic applications the modification of an active pharmaceutical oxindole to prepare a collection of perfluorinated analogs can be highlighted.

**Scheme 15:**
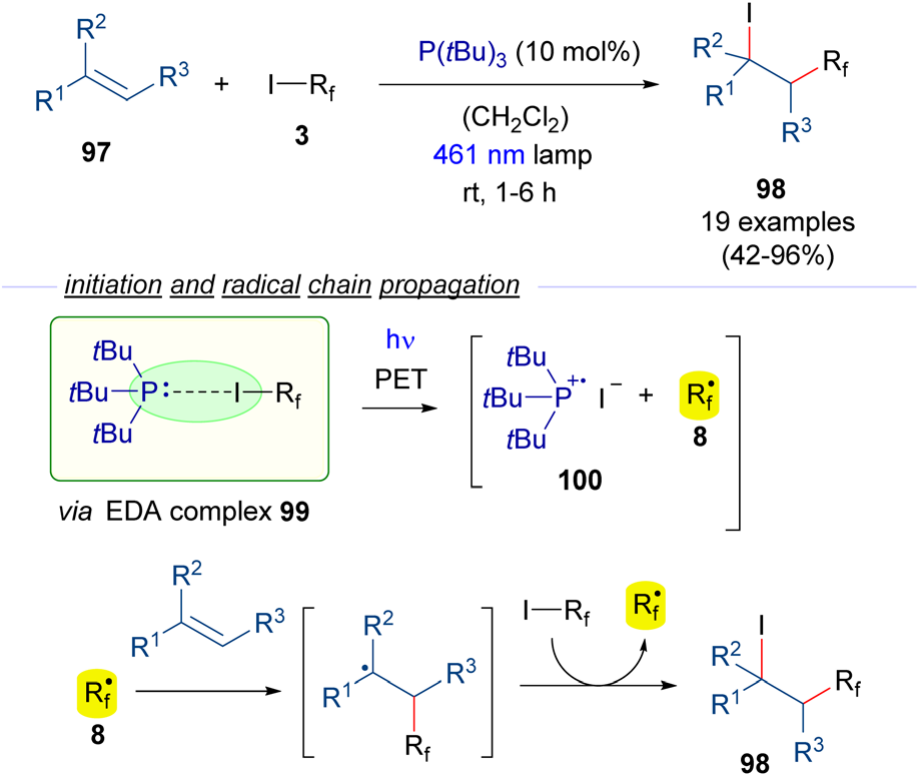
Photochemical HB-assisted catalysis with P(*t*Bu)_3_ for ATRA reactions of olefins.

The photochemical generation of perfluoroalkyl radicals from perfluoroalkyl iodides can also be promoted by phosphines as HB acceptors (Scheme 15).^39^ In this regard, the group of Czekelius reported in 2019 a new protocol to accomplish the 1,2-iodoperfluoroalkylation of alkenes 97 using *t*Bu_3_P as the promoter for the formation of the perfluoroalkyl radical. Thus, irradiation with blue light of the EDA complex formed between the phosphine and the R_f_–I 99 induces its fragmentation into the radical species 100 and 8. The formation of the complex 99 is supported by ^19^F-NMR titration experiments and UV-vis analysis. Then, the radical chain reaction as previously described (Scheme 9) provides the iodo perfluoroalkylated alkane 98. Importantly, in this process the phosphine, which acts only in the initiation step, can be used in a substoichiometric amount.

**Scheme 16:**
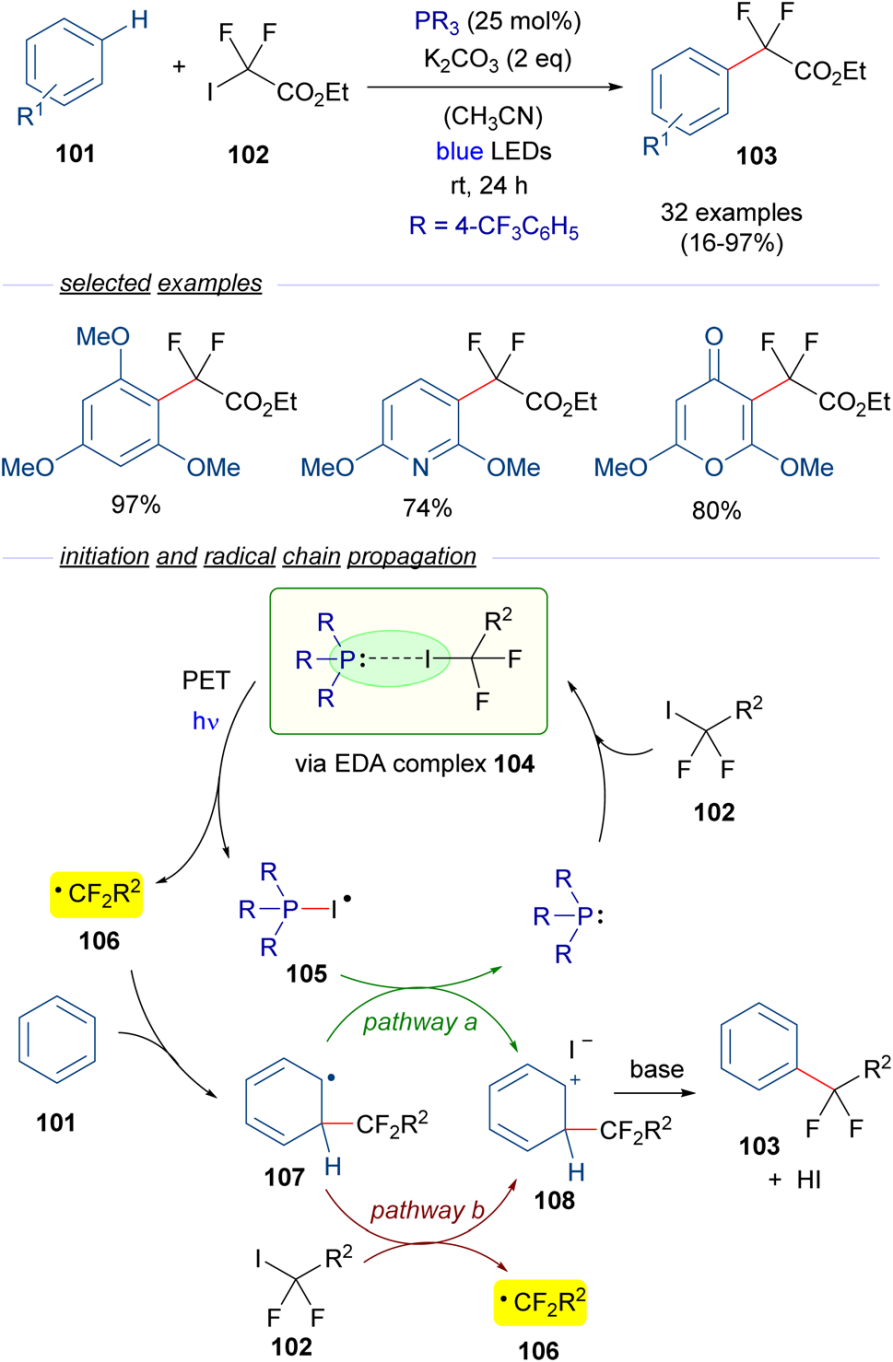
Difluoroalkylation reactions of arenes 101 mediated by photoexcitation of phosphine-containing HB complexes.

A year later, in 2020, Zhang and coworkers reported a visible-light promoted phosphine-catalyzed difluoroalkylation reaction of arenes 101 (Scheme 16).^40^ This time, an EDA complex 104 is formed between P(4-CF_3_C_6_H_5_)_3_ and difluoroalkyl iodide 102. The formation of this complex was examined and supported through NMR analysis and the identification of a CT band in the UV-vis spectra. Photoinduced fragmentation of this complex forms the radical species 105 and the difluoroalkyl radical 106, which adds to the arene 101 to form a new radical intermediate 107. At this point, two different pathways may operate. Pathway a involves an oxidation of the latter radical 107 by 105, what regenerates the phosphine and provides the carbocation intermediate 108. This intermediate can also be accessed through pathway b, in which the radical intermediate 107 reduces an equivalent of the difluoroalkyl iodide 102 starting a radical chain propagation by regenerating the radical species 106. This proposal is consistent with the determined quantum yield for this transformation (*φ* = 2.4). Eventually, deprotonation mediated by the base of 108 causes rearomatization and furnishes the final difluoroalkylated arenes 103. In summary, a novel protocol for the difluoromethylation of unactivated arenes was developed, in which a broad range of substrates could be accessed including several pharmaceutical agents.

**Scheme 17:**
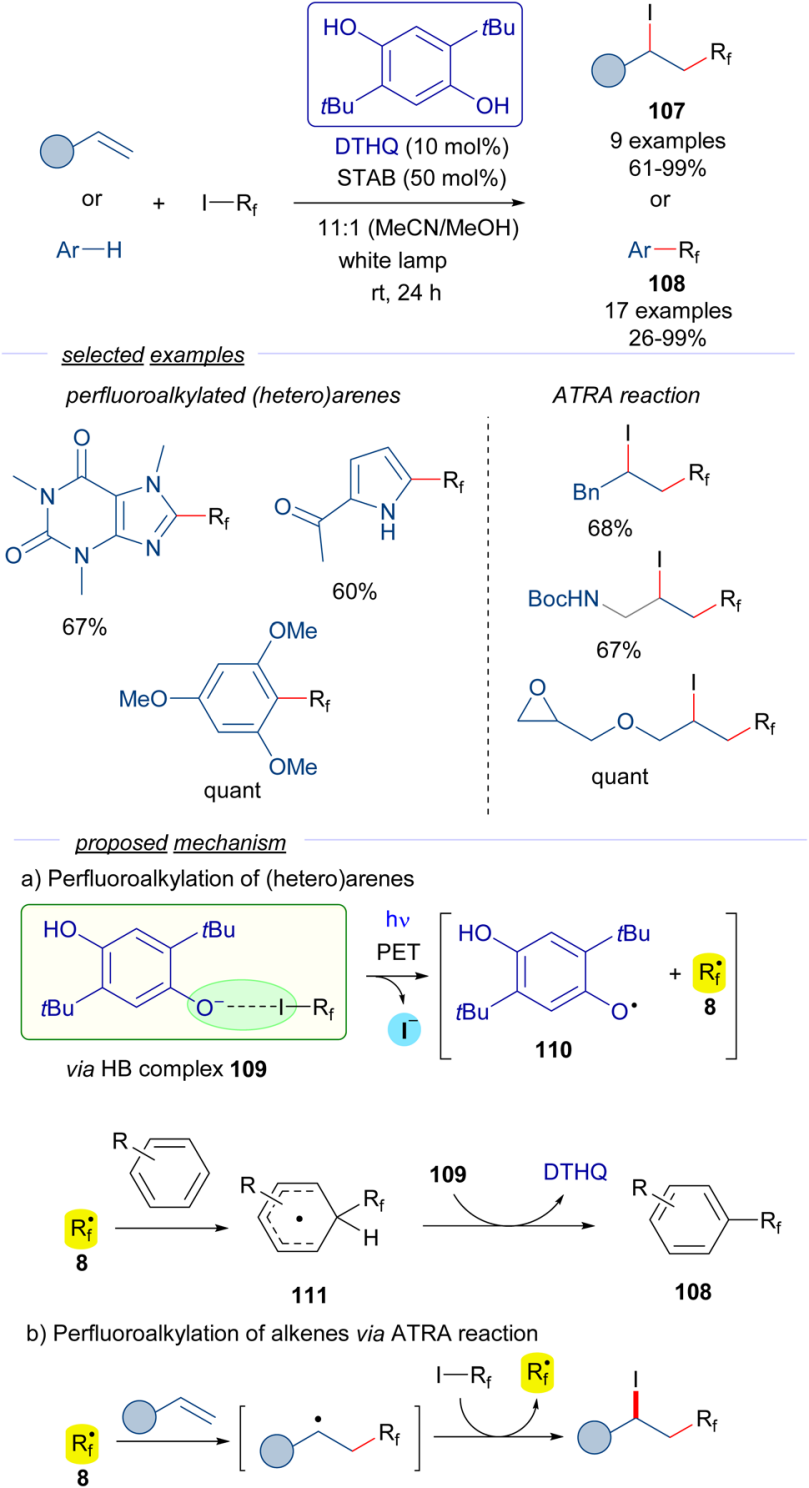
Photochemical HB-assisted catalysis with DTHQ. STAB: sodium triacetoxyborohydride.

In a recent contribution reported by Pitre,^41^ 2,5-di-*t*Bu-hydroquinone DTHQ is employed as a HB acceptor to carry out the perfluoroalkylation of both (hetero)arenes and the iodoperfluoroalkylation of olefins *via* ATRA reactions (Scheme 17).

The reaction is initiated by photoexcitation of the HB complex 109, formed by DTHQ phenoxide and perfluoroalkyl iodide 3. The existence of this complex is supported by DFT calculations and the analysis of UV-vis spectra, where a new CT band was observed. Fragmentation leads to the perfluoroalkyl radical 8, the DTHQ phenoxide radical 110, and iodide. In the perfluoroalkylation of heteroarenes, radical 8 adds to the (hetero)arene, forming a new radical intermediate 111. Hydrogen atom abstraction by DTHQ phenoxide radical 110 regenerates the DTHQ catalyst and enables releasing the perfluoroalkylated arenes 108. On the other hand, the ATRA reaction of the perfluoroalkyl radical 8 to the olefins follows the radical chain mechanism previously discussed in Schemes 9 and 15. Overall, these transformations exhibit an unprecedented utility of HB complexes to generate oxygen-centered radicals in a catalytic manner, which found broad application in the synthesis of perfluoroalkylated (heteroarenes) 108 and perfluoroalkyl halides 107.

##### 2.3.1.3 Mode B: radical recombination

**Scheme 18:**
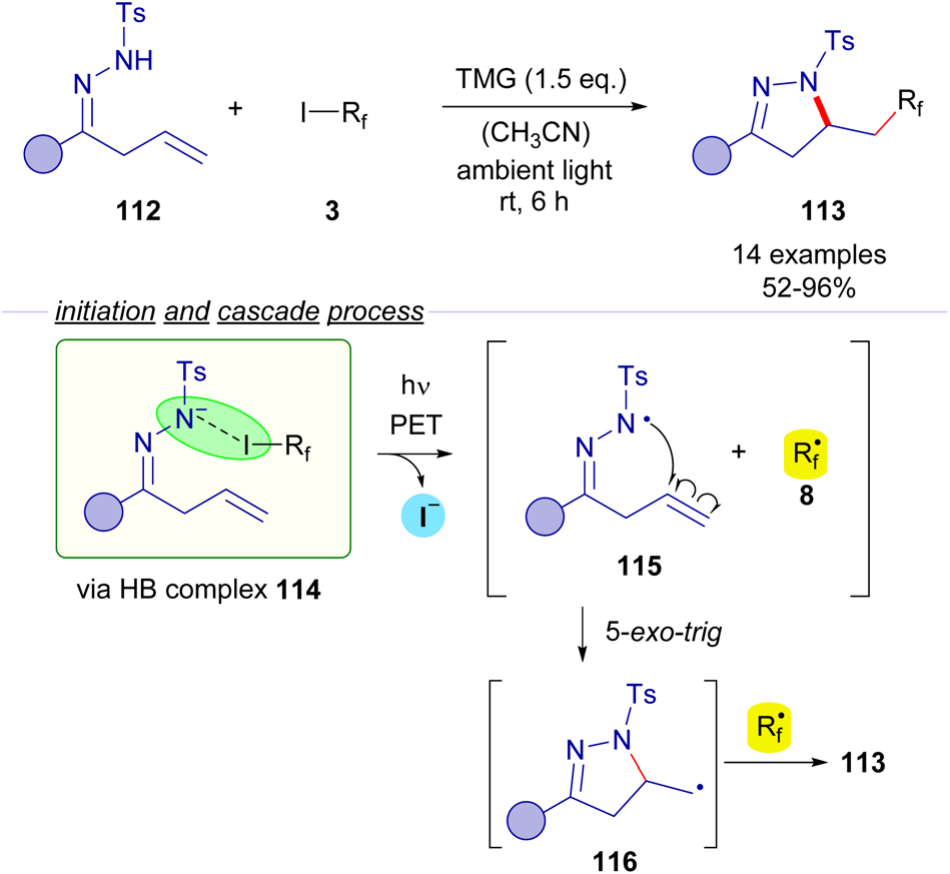
Synthesis of pyrazolines 113 by photochemical perfluoroalkylative cyclization of β,γ-unsaturated hydrazones 112.

In 2017, Cai and coworkers reported a photochemical halogen-bonding assisted cascade cyclization reaction employing β,γ-unsaturated *N*-tosylhydrazones 112 and perfluoroalkyl iodides 3 for the formation of pyrazolines 113 (Scheme 18).^42^ A coloured HB adduct was formed when mixing both coupling partners with TMG as the base. Excitation with ambient light of the HB complex 114 caused fragmentation of the perfluoroalkyl iodide, generating an *N*-centered radical 115, which experiences a fast intramolecular 5-*exo-trig* cyclization to form a primary alkyl radical species 116 that is subsequently combined with the perfluoroalkyl radical intermediate 8. The transformation was extended for the synthesis of an array of different pyrazolines 113 with good yields and exhibiting a good functional group tolerance.

**Scheme 19:**
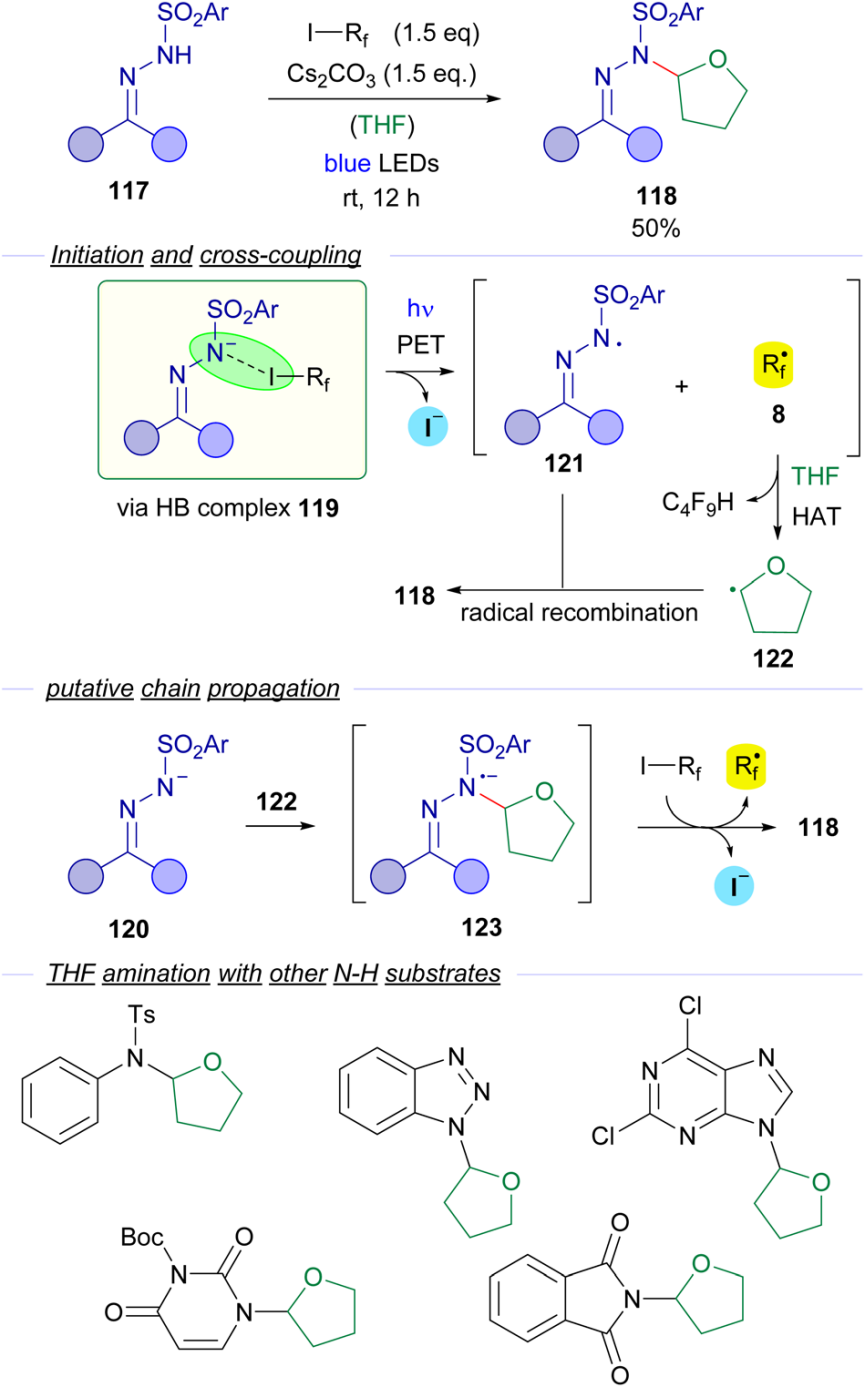
Visible light promoted α-C–H amination of ethers.

*N*-sulfonylhydrazones were also employed by Cheng and coworkers is 2018 in a visible light halogen-bond-promoted α-C–H amination of ethers and thioethers (Scheme 19).^43^ The existence of a halogen-bonding complex 119 between the hydrazone anion 120 and the perfluoroalkyl iodide is supported by ^19^F-NMR titration experiments and a UV-vis spectra analysis. It is worth mentioning that apart from photochemical initiation, the reaction also proceeded well upon thermal activation. The irradiation of the halogen bonding complex 119 would initiate the transformation, generating hydrazonyl radical 121 and perfluoroalkyl radical 8. Radical 8 participates in a hydrogen atom transfer (HAT) event with a molecule of the THF solvent generating the α-oxyradical 122 that recombines with the hydrazonyl radical 121 to give the coupling product 118. The authors suggest that a radical chain propagation might also be operating. The recombination of α-oxyradical 122 with hydrazonyl anion 120 would produce the radical anion intermediate 123, which could reduce another molecule of 3, starting the radical propagation *via* delivery of a new equivalent of the radical species 8. The authors also explained that the cleavage of the C(sp^3^)–H bond is the rate limiting step of this reaction, since a significant kinetic isotopic effect was observed (*K*_H_/*K*_D_ = 3). The reaction could be applied with an array of *N*-sulfonylhydrazones derived from cyclic and acyclic ketones. Moreover, the C–H amination reaction is not restricted to the use of *N*-sulfonylhydrazones. In fact, the amination of THF could also be performed with other N–H compounds such as tosylamides and N–H free heterocycles.

**Scheme 20:**
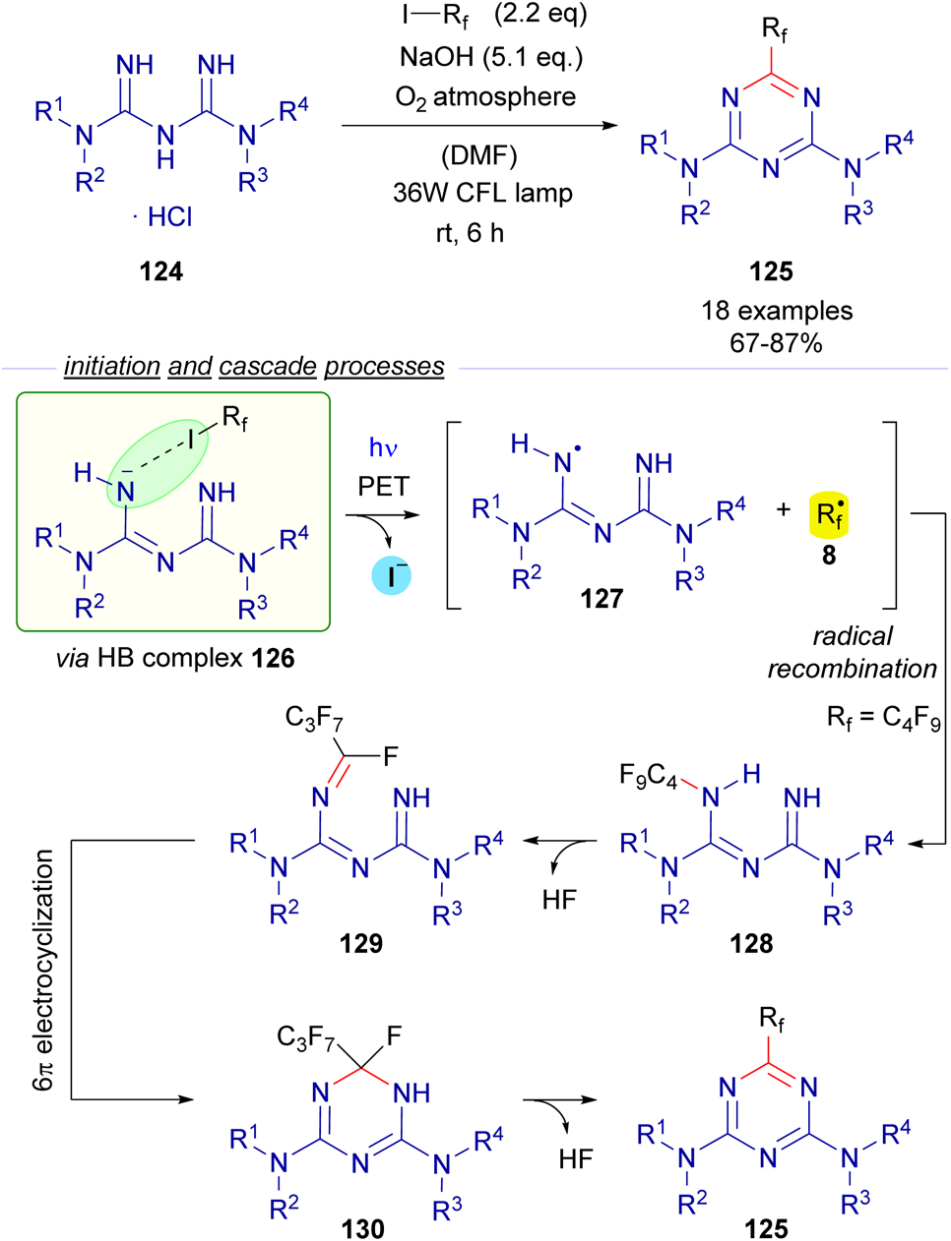
Synthesis of perfluoroalkylated triazines 125*via* photochemical activation of the HB complex 126.

Also in 2019, Ren and Liang described an unusual photochemical [5 + 1] annulation reaction for the synthesis of perfluoroalkylated triazines 125 from biguanidines 124 (Scheme 20).^44^ The reaction is initiated by excitation with white visible light of the HB complex 126 between biguanidine and the perfluoroalkyl iodide, which undergoes fragmentation and creates the two different radical species 127 and 8. Direct radical recombination of these species produces the perfluoroalkylated biguanidine 128, which after elimination of HF forms the triazatriene 129. A 6π electrocyclization would yield the dihydrotriazine 130, which after elimination of HF delivers the final perfluotoalkylated triazines 125. The examination of the UV-vis absorption profiles revealed the characteristic CT-band of the HB complex 126. Mechanistic studies oriented to support the radical pathway involved a quantum yield determination (*φ* = 0.03), which excluded radical chain propagation processes and inhibition of the reaction by the radical scavengers *p*-dinitrobenzene and TEMPO.

**Scheme 21:**
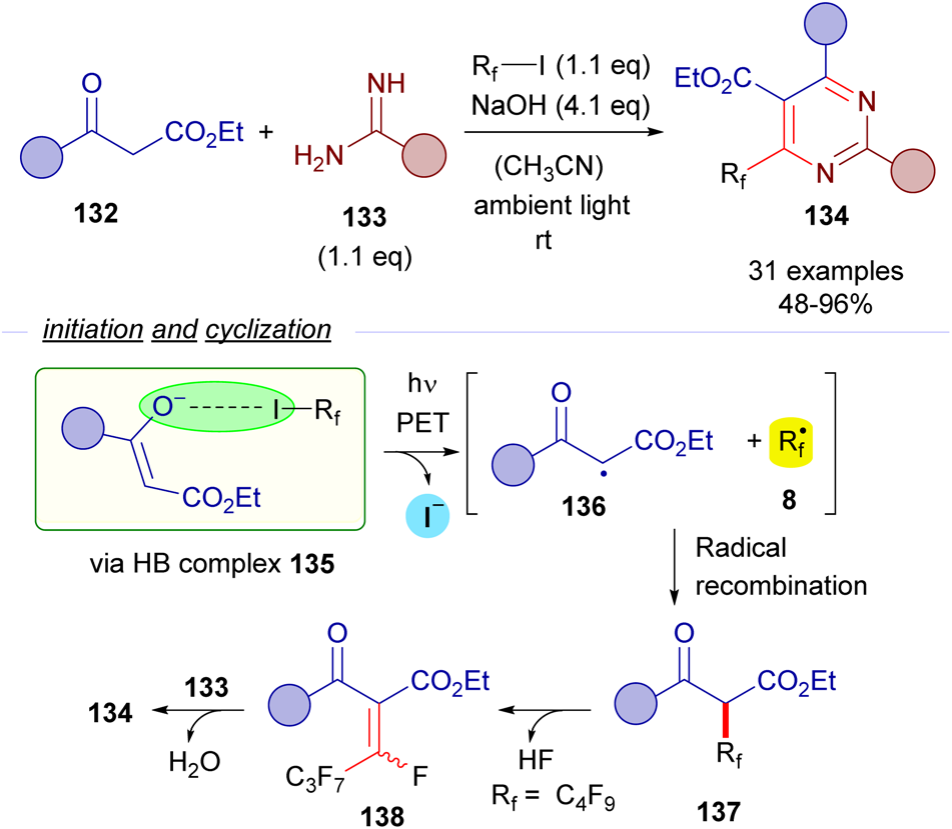
Multicomponent halogen-bonding-initiated synthesis of pyrimidines 134.

The formation of perfluoroalkyl radicals *via* photoexcitation of EDA complexes held by a O–X halogen bond has been exploited by Liang and Bi in an ambient-light-initiated multicomponent synthesis of perfluoroalkylated pyrimidines from ketoesters 132, amidines or guanidines and perfluoroalkyl iodides (Scheme 21).^45^ Similarly to some of the seminal examples reported by Melchiorre, a ketoester enolate is responsible for the formation of a halogen bonding complex 135 with a perfluoroalkyl iodide. DFT calculations run by the authors supported the formation of this complex. The photoexcitation of this adduct with ambient light promotes a fragmentation and the generation of radicals 136 and 8. A fast radical recombination would produce the perfluoroalkylated ketoester 137, which experiences elimination of HF to form the α,β-unsaturated ketoester 138. Finally, a heterocyclization reaction combining 138 and the guanidines or amidines 133 enabled the formation of the pyrimidines 134.

**Scheme 22:**
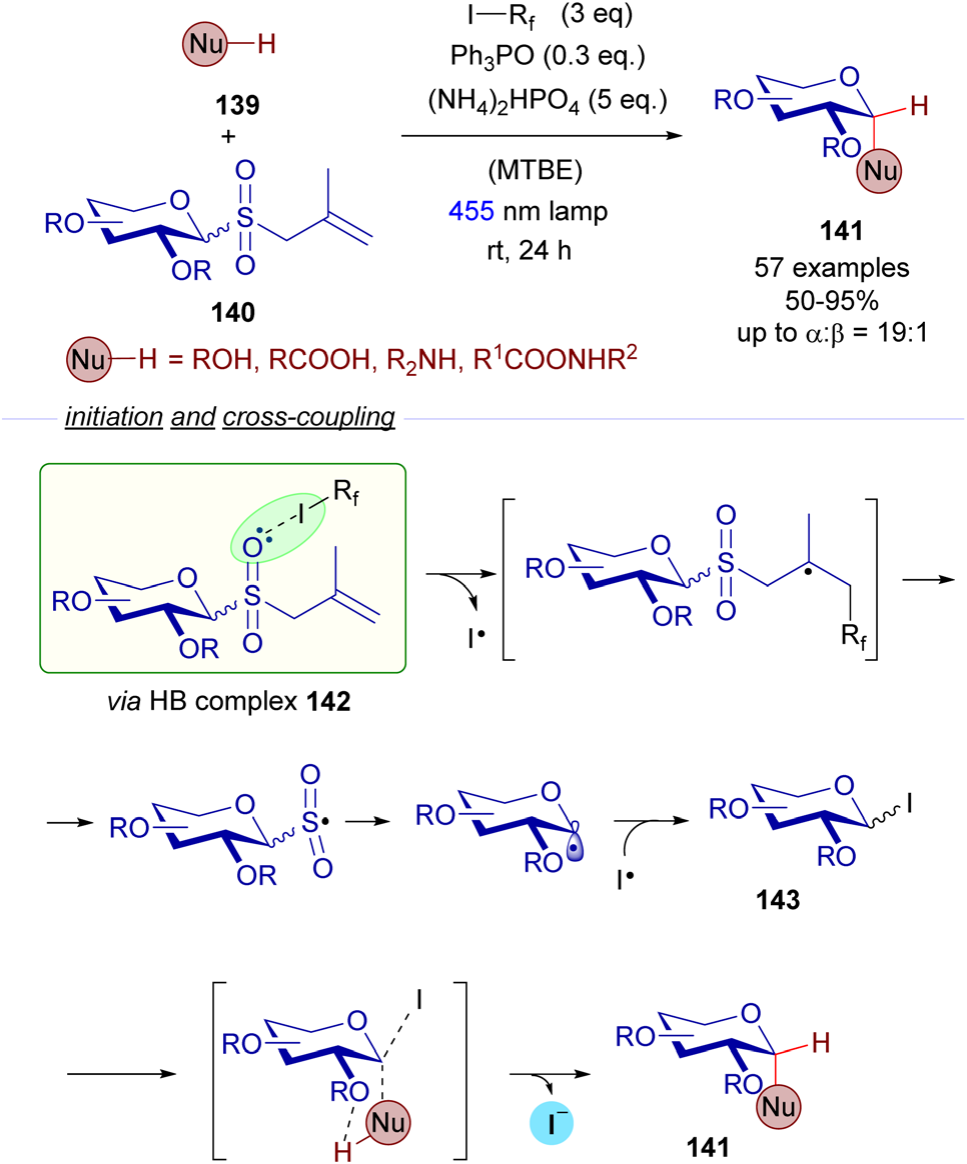
Halogen-bond-assisted radical activation of glycosyl donors 140 for the formation of 1,2-*cis*-disubstituted glycosides 141. MTBE: methyl *tert*-butyl ether.

A relevant recent example dealing with the photochemical fragmentation of perfluoroalkyl iodides *via* HB complexes corresponds to the work reported in 2022 by the research groups of Houk and Niu on the stereoselective synthesis of 1,2-*cis*-glycosydes 141 from allyl glycosyl sulfones 140 (Scheme 22).^46^ This time, the halogen-bonding adduct 142 which was excited with visible light was formed as a result of the interaction of allyl glycosyl sulfones 140 (HB acceptor) and perfluoroalkyliodides (HB donor). Mechanistic studies were run to identify this HB complex 142 (^19^F-NMR titrations and DFT calculations). Photoinduced fragmentation of this complex through a cascade of radical reactions forms the electrophilic glycosyl iodide 143 that can be trapped by nucleophiles. Apart from the halogen-bonding activation mode, this reaction relies additionally on hydrogen-bonding interactions as depicted in Scheme 22 between the external nucleophile 139 and the ether residue at C_2_ of the sugar moiety. These interactions are responsible for the stereoselectivity observed in the reaction, where both substitutions present at C_1_ and C_2_ of the final glycoside 141 are predominantly in a *cis* arrangement. The reaction tolerated different nucleophilic species such as alcohols, acids, amines and amides, showcasing a broad functional group tolerance. Thus, this reaction features a dual activation mode: both a photochemical halogen-bonding assisted generation of glycosyl radicals and hydrogen-bonding interactions, which provide the transformation with high levels of stereoselectivity. This radical cascade process represents a sustainable alternative to other conventional acid-promoted polar reactions for the creation of 1,2-*cis*-glycosidic bonds.

#### 2.3.2 Functionalized C sp^3^-centered radicals

The chemistry presented until this point shows that the highly electrophilic perfluoroalkyl iodides 3 are excellent halogen-bond donors for EDA or HB-complex driven photochemical transformations. Nevertheless, there are other examples in which alkyl halides have been employed as HB donors in photochemical catalyst-free reactions as precursors of alkyl C sp^3^-centered radicals.

##### 2.3.2.1 Radical chain reactions Mode A.1

**Scheme 23:**
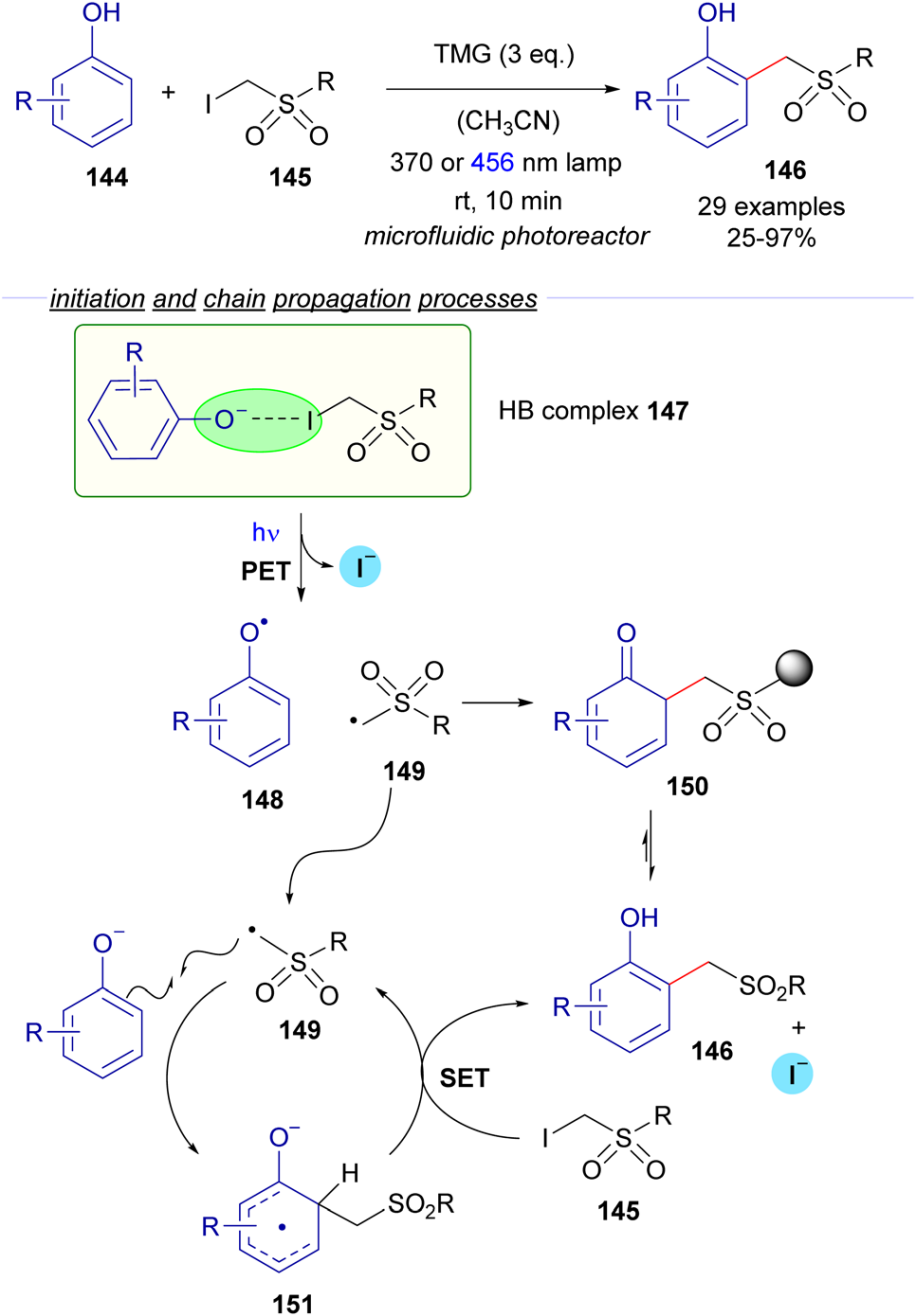
Photochemical halogen-bonding assisted cross-coupling between phenols 144 and α-iodosulfones 145.

In 2022, the research group of Dell'Amico reported a flow protocol for the photochemical cross-coupling reaction of phenols 144 and α-iodosulfones 145 (Scheme 23).^47^ The transformation is initiated by the deprotonation of the phenols mediated by an organic base (DBU). The interaction between the phenoxide and the iodosufone provides the halogen-bonding complex 147, the existence of which was validated through UV-vis spectra analysis and DFT calculations. The photoexcitation of 147 with different light sources (370 or 456 nm lamp) generates the radical species 148 and 149, the recombination of which might lead to the formation of the *ortho*-alkylated phenols 146 after rearomatization from the intermediate 150. However, the authors determined that the quantum yield of the transformation is above the unit (*φ* = 2), which is indicative of a radical chain process. Thus, a propagation was proposed to occur as depicted in Scheme 23. First, radical addition of 149 to the phenoxide would produce the radical anion 151, which is postulated to react with iodosulfone 145 in a SET event regenerating the radical intermediate 149 with the concomitant formation of the final product 146. To support this proposal, the redox potential of α-iodosulfone 145 was measured through cyclic voltammetry (*E*_red_ (145/145^˙−^) = −1.4 V *vs.* SCE in MeCN). The relatively low redox potential of 145 suggests that the reduction of 145 by the SET process from 151 is a feasible process, giving support to the radical chain mechanism. Additional features of this transformation are its application for the structural modification of biologically active molecules (such as tyrosine and paracetamol) and the consecutive access to *ortho*-methylated phenols upon reductive cleavage of the sulfone moiety.

##### 2.3.2.2 Radical chain reactions Mode A.2

**Scheme 24:**
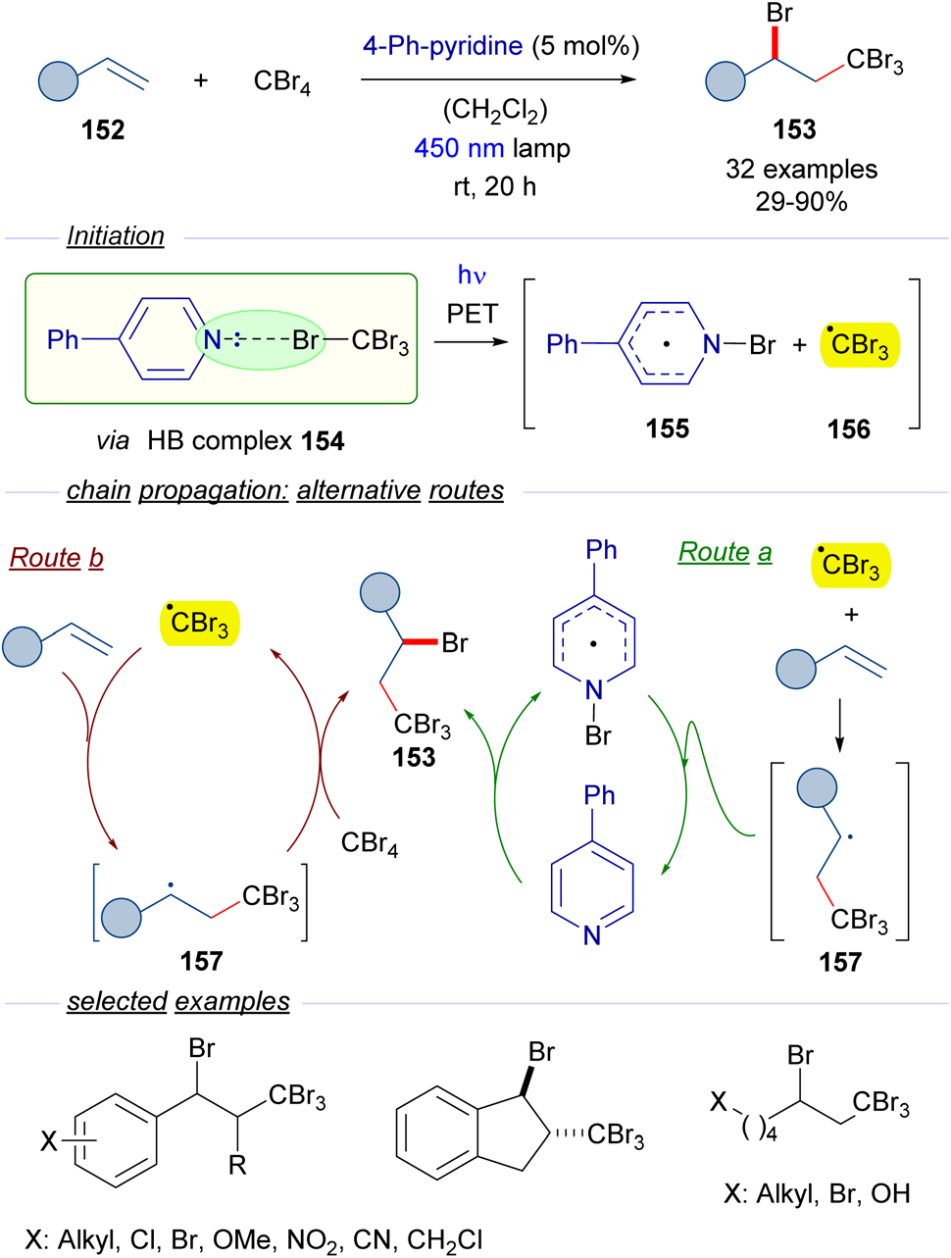
ATRA reaction of olefins through the formation of a HB complex 154 with catalytic amounts of 4-phenylpyridine.

In 2020, Yamaguchi and Itoh reported a protocol for the photochemical activation of CBr_4_ through the formation of a halogen-bonding complex with catalytic amounts of 4-phenylpyridine as a HB acceptor (Scheme 24).^48^ The reactions in the presence of olefins 152 give rise to the ATRA products 153 with the incorporation of Br and CBr_3_ in the vicinal carbons of the alkene. The transformation is started by the photoexcitation of the HB complex 154 with blue light, which after fragmentation furnishes the *N*-bromopyridyl radical 155 and the radical cation CBr_3_ radical 156. The formation of this HB complex was identified through the observation of a new CT band in the UV-vis analysis. At this point two different routes are proposed by the authors. In route a, an ATRA reaction occurs due to the addition of tribromomethyl radical 156 to the olefin 152, which produces a new radical intermediate 157. This radical abstracts the bromine from *N*-bromopyridyl radical 155 to regenerate the catalyst 4-phenylpyridine and at the same time forms the final product 153. An alternative pathway (route b) would involve the reaction of the radical 157 with a new molecule of CBr_4_ to start a radical propagation by forming the tribromomethyl radical and the 1,2-disubstituted olefin 153. The radical nature of this transformation was supported by the trapping of radical intermediates by the radical scavenger TEMPO. All in all, this reaction represents a novel metal-free activation of tetrabromomethane in which the HB acceptor is used in a catalytic fashion. From the synthetic point of view, the reaction can be seen as the insertion of the alkene in the C–Br bond through stepwise radical reactions. This transformation has been applied to substituted styrenes, internal olefins such as indene and alkyl substituted olefins, among others.

**Scheme 25:**
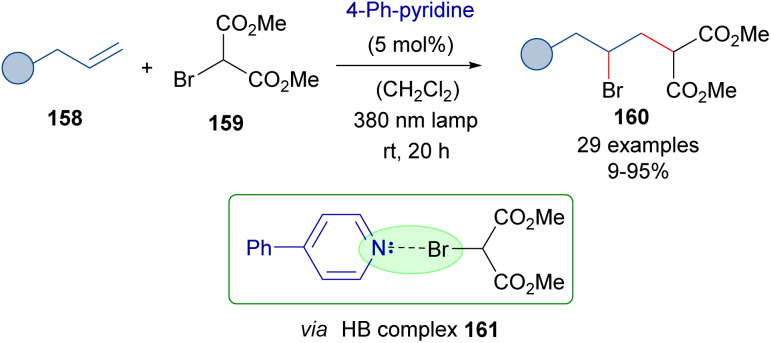
ATRA reaction of olefins with bromomalonates 159 through the formation of a HB complex 161 and catalytic amounts of 4-phenylpyridine.

As a further application of the photochemical activation of HB complexes employing catalytic amounts of an 4-phenylpyridine, the same authors reported in 2021 the photochemical activation of bromomalonates 159 and their reaction with alkenes to provide the γ-bromomalonates 160 (Scheme 25).^49^ A mechanism analogous to that of Scheme 24 was proposed.

**Scheme 26:**
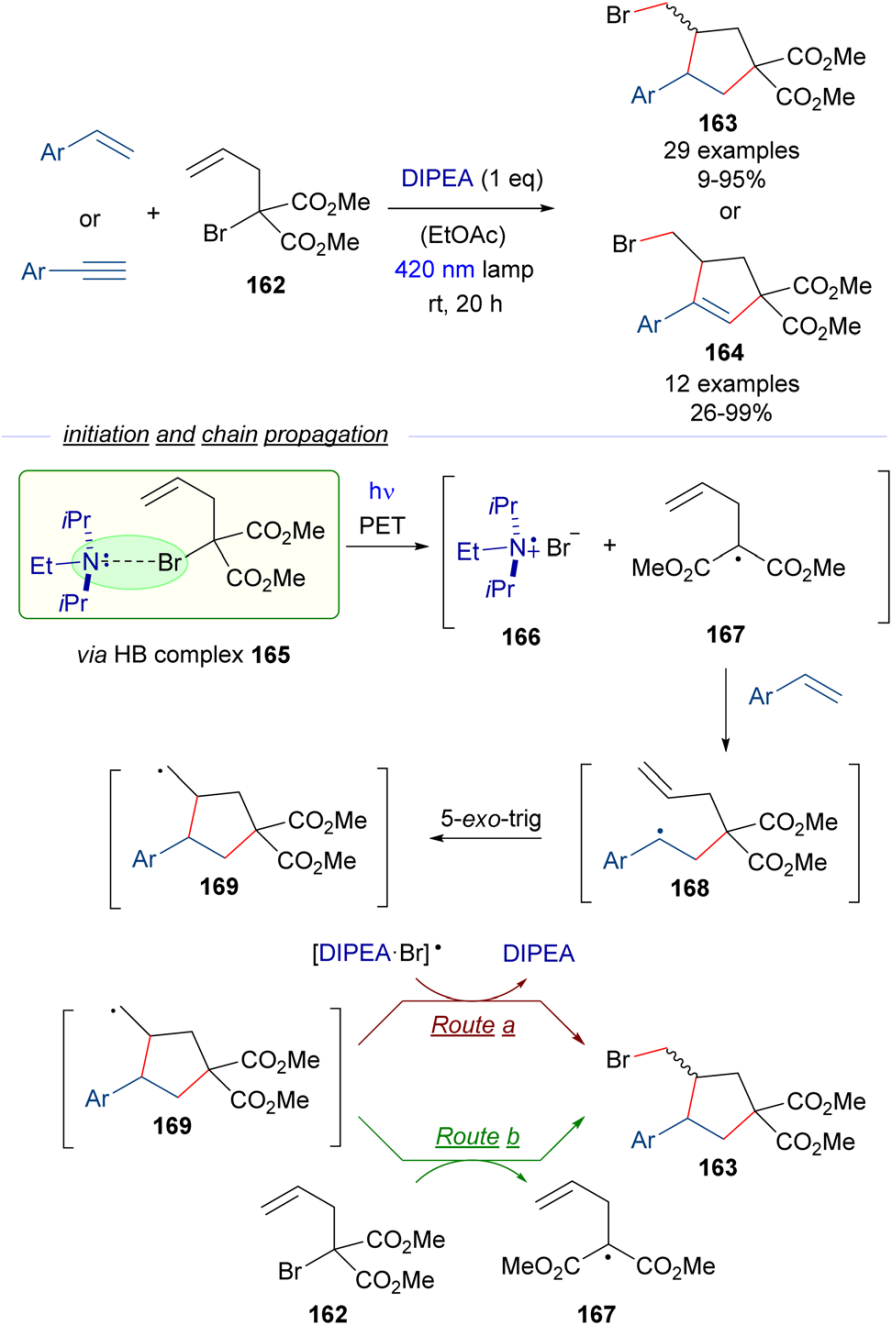
ATRA/ATRC reaction of α-allyl-α-bromomalonate 162 with styrenes and arylacetylenes for the construction of functionalized cyclopentanes and cyclopentenes 163 and 164.

To extend the synthetic utility of the HB activation of α-bromomalonates, Yamaguchi and Itoh reported also in 2021 a radical cascade cyclization employing α-allyl-α-bromomalonate 162 and styrenes or arylacetylenes as starting materials for the synthesis of functionalized cyclopentanes 163 and cyclopentenes 164 through an atom-transfer radical addition/atom-transfer radical cyclization (ATRA/ATRC) protocol (Scheme 26).^50^ In this case a stoichiometric amount of DIPEA was used as the promoter for the generation of the initial radical through the HB-complex formation/PET/fragmentation sequence. Thus, upon photoinduced fragmentation of the HB complex 165 the radical intermediates 166 and 167 are proposed to be formed. The malonate radical 167 adds to the olefin, forming the radical intermediate 168, that undergoes 5-*exo-trig* cyclization to form the new radical 169, which finally abstracts the bromine atom from the [DIPEA·Br] radical 166 (route a) or from the bromomalonate 162 (route b) to complete the radical chain reaction. The step-economy of this protocol is shown in the modular formation of two C sp^3^–C sp^2^ bonds and one C sp^3^–X bond in a single synthetic step.

**Scheme 27:**
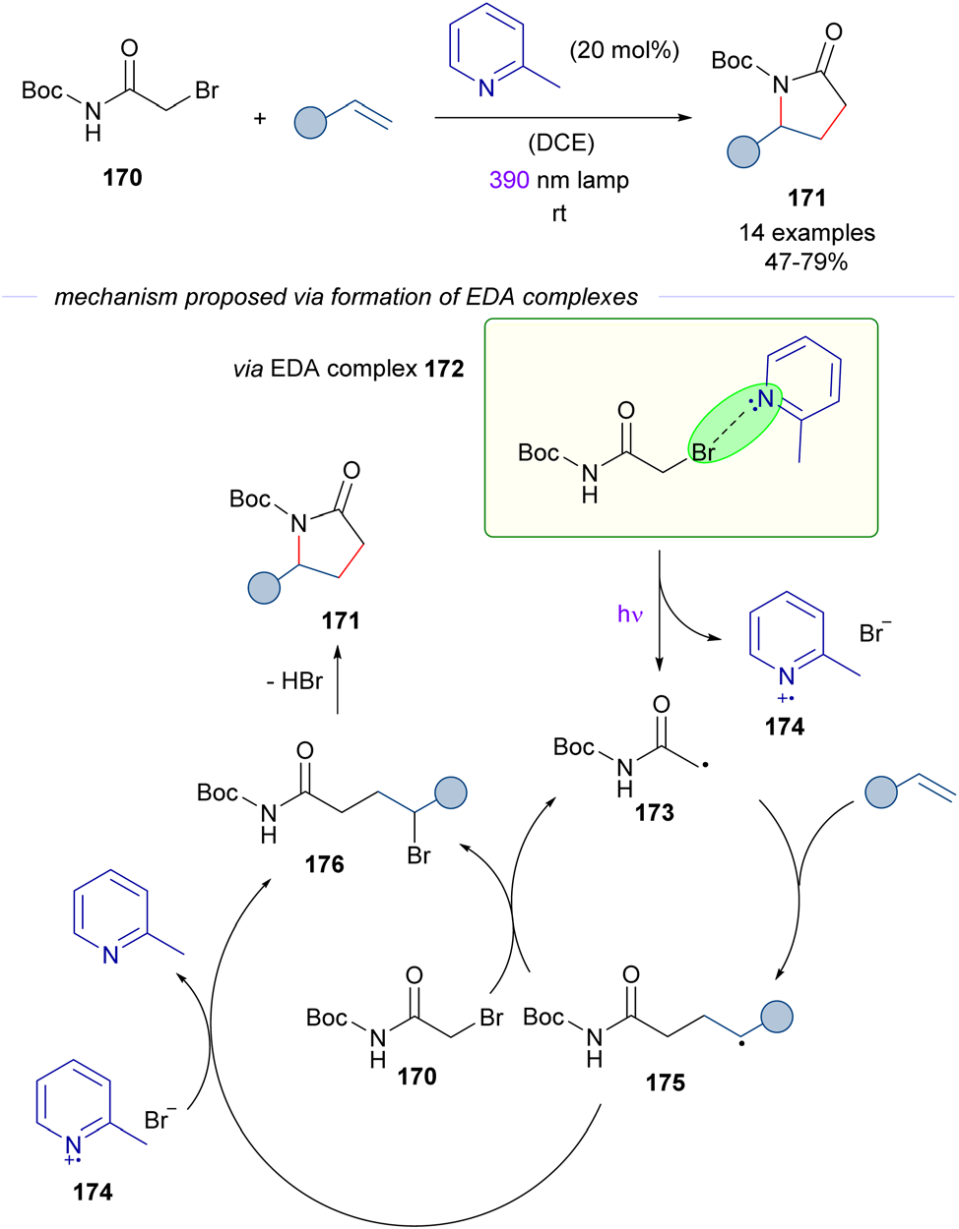
Light-driven excitation of EDA complexes 172 as the key to synthesize γ-lactams 171.

The activation of alkyl bromides by organic Lewis bases was applied in 2023 by Rovis and co-workers^51^ in a novel approach to γ-lactams 171 from α-bromoacetamides 170 and alkenes (Scheme 27). During the study of the photocatalytic reaction, it was discovered that the cascade process occurred also in the absence of a photocatalyst through an EDA complex photoactivation. The EDA based transformation is similar to that described in Schemes 23–25 and is initiated by excitation with purple light of the EDA complex 172, which is assembled by the interaction of 2-methylpyridine and α-bromoamide 170. This complex was identified through UV-vis analysis. The C sp^3^-centered radical 173 adds to the olefin, generating the electron-rich radical intermediate 175. At this point, a halogen atom transfer (XAT) from α-bromoacetamide 170 or from the 2-MePy·Br radical 174 furnishes the γ-brominated amide 176 and regenerates the radical 173 or the pyridine base respectively. The experimental determination of the quantum yield (*φ* = 14) supported the radical chain mechanism. In summary, the transformation represents a novel [3 + 2] entry to γ-lactams that overcomes previous limitations of redox approaches to the synthesis of these compounds, such as the necessity to preinstall electron-withdrawing groups at the nitrogen atom of the amide to enhance the electrophilicity of the corresponding radical intermediates. It is also worth noting that in this case the activation of the alkyl bromide does not require strong electron-withdrawing substituents. In connection with this transformation, the photochemical activation of the C–Br bond on α-bromoacetonitrile and α-bromoesters in the presence of 2,6-dimethylpyridine has been reported by Trapp.^52^ These reactions are not discussed herein because an alternative mechanism which does not involve a halogen-bond EDA complex has been proposed. Nevertheless, readers are encouraged to revise the alternative mechanistic proposal.

**Scheme 28:**
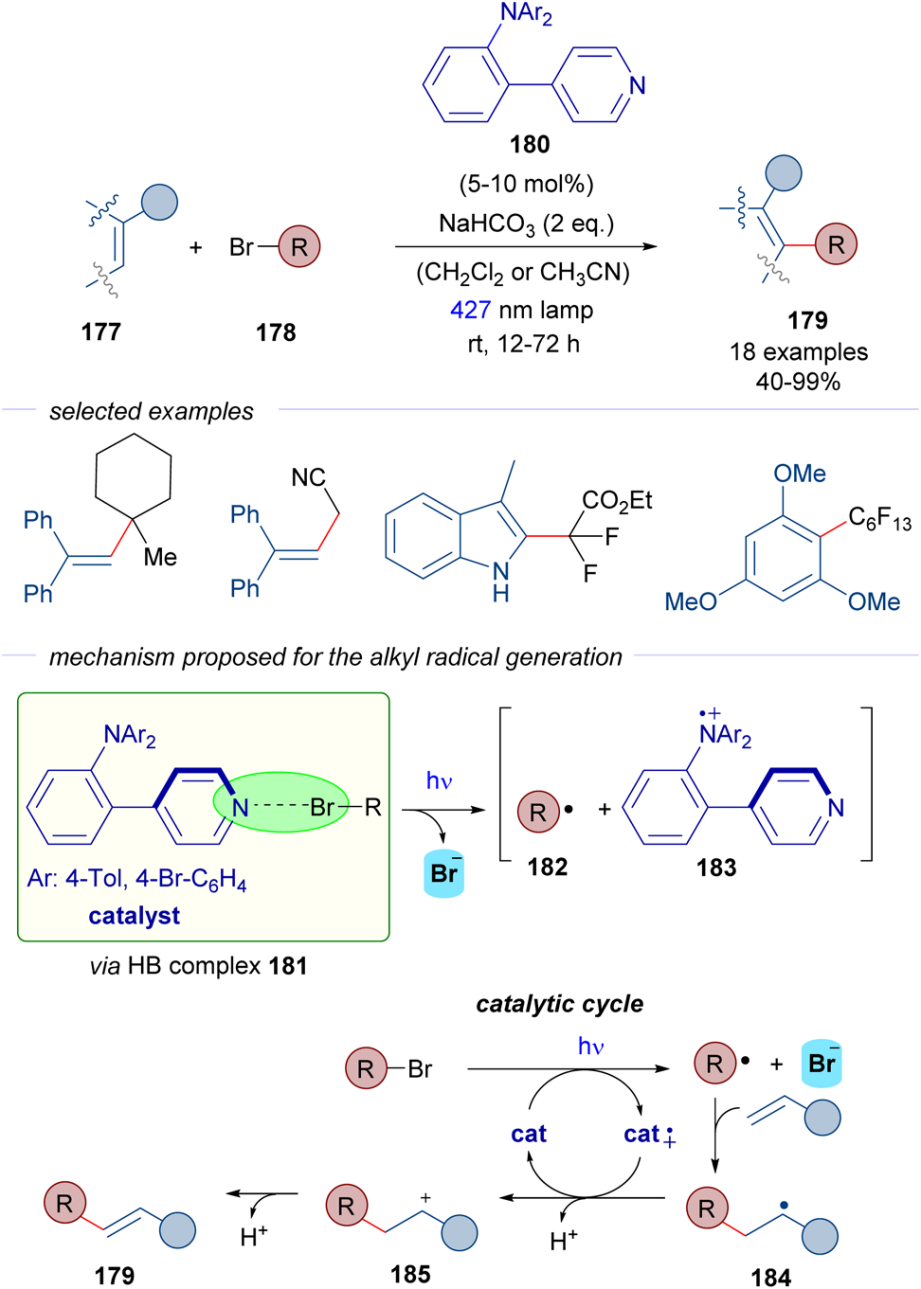
C–H alkylation of alkenes by the direct generation of radicals from alkyl bromides 178 through halogen bonding *via* a pyridine-based donor–acceptor photocatalyst 180.

In 2022, Nanjo and Takemoto described the C–H alkylation of alkenes 177 through a catalytic strategy that involves the generation of C sp^3^-centered radicals from alkyl bromides 178 (Scheme 28).^53^ A pyridine-based scaffold 180 was designed and synthesized with the objective of generating halogen-bonding adducts with the alkyl bromides. Upon photoexcitation of the HB complex 181, the alkyl centered radicals 182 would be formed and further trapped with different electron rich olefins and aromatic systems as radical acceptors to produce radical 184. Unlike the examples described before, the reaction product does not incorporate the bromine. This time, the radical cation of the Lewis base catalyst 183 is able to oxidize the radical 184 into the corresponding cation 185, which upon loss of a proton delivers the final products, to form an array of structurally diverse C–H alkylated alkenes and arenes 179.

The reaction is remarkable with regard to the scope of the bromides, since unactivated tertiary alkyl halides and also tertiary and secondary alkyl halides with electron-withdrawing substituents are compatible with the reaction.

According to the authors' proposal, the successful design of the pyridine catalyst 180 relies on the donor–acceptor character. The pyridine can act as a Lewis base in the formation of the HB-complex 181. Moreover, the presence of the triarylamino moiety enables the existence of a persistent radical cation 183, which acts as an oxidant in the catalytic cycle. Thus, in this case a new mechanism is proposed where the HB-bond acceptor acts also as a photoredox catalyst. Detailed mechanistic studies were carried out to support the mechanistic proposal: detection of the halogen-bonding complex 181 by DOSY ^1^H-NMR experiments, radical trapping of radical intermediates by TEMPO, inhibition of the reaction by superstoichiometric amounts of halide anions such as Cl^−^ or Br^−^, light on-off experiments and quantum yield measurements that excluded the possibility of a radical chain reaction. All in all, this transformation represents a remarkable example of a photochemical halogen-bonding initiated reaction that uses a halogen-bond acceptor photocatalyst, which can generate C sp^3^-centered radicals from both activated and non-activated alkyl halides.

### 2.4. Formation of aromatic C sp^2^ centered radicals

The formation of aryl-centered radicals is a topic of great interest in synthetic organic chemistry.^54^ Very recent contributions have shown that the homolytic cleavage of the C_Ar_–X bond to produce aryl radicals can be achieved through the photochemical activation of HB or EDA complexes. Again, examples of the different general modes of reactivity can be found and therefore this section is organized accordingly.

#### 2.4.1 Mode A: radical chain reactions

**Scheme 29:**
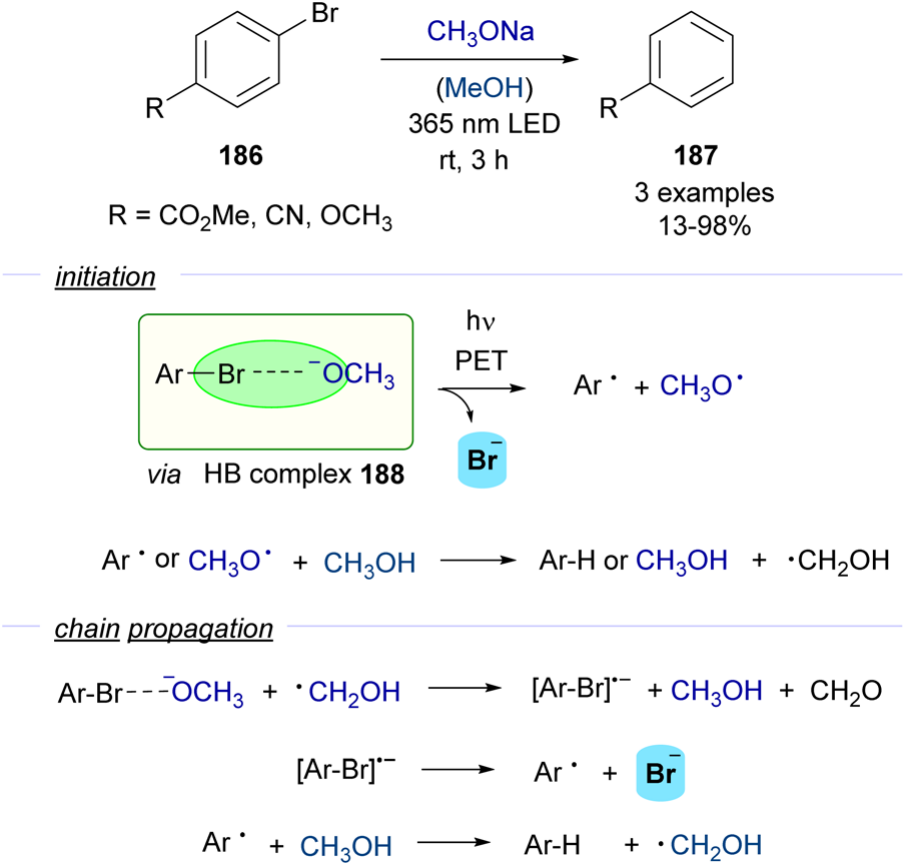
Photoinduced dehalogenation of *p*-substituted halobenzenes 186 in the presence of a base and methanol.

One of the first contributions which described the formation of aryl radicals *via* halogen-bonding photochemical activation was reported in 2019 by Scaiano, Lanterna and co-workers (Scheme 29).^55^

A photochemical dehalogenation of aryl halides was achieved *via* activation of a HB complex that was formed between aryl bromides 186 and methoxide with low-energy UV light. The photochemical activity of this complex 188 was supported by DFT calculations, which suggested that the formation of the HB complex weakened the C–Br bond facilitating its cleavage. The formation of the HB complex 188 was rationalized through UV-vis analysis. The inhibition of the reaction upon addition of TEMPO as a radical scavenger supported the existence of a radical pathway. Quantum yield determinations (*φ* = 43) indicated that the reaction should proceed through a radical chain propagation mechanism. A rather complex radical chain mechanism was hypothesized to explain the experimental observations (Scheme 29). If the proposed mechanism were operative, stoichiometric amounts of formaldehyde would be generated in the propagation steps, which indeed could be detected by analytical methods. Although the scope of this transformation was very limited, it served as a proof of concept for the possibility to generate aryl-centered radicals through photochemical halogen-bonding activation of aryl halides.

#### 2.4.2 Mode A.2: radical chain reactions with an external promoter

**Scheme 30:**
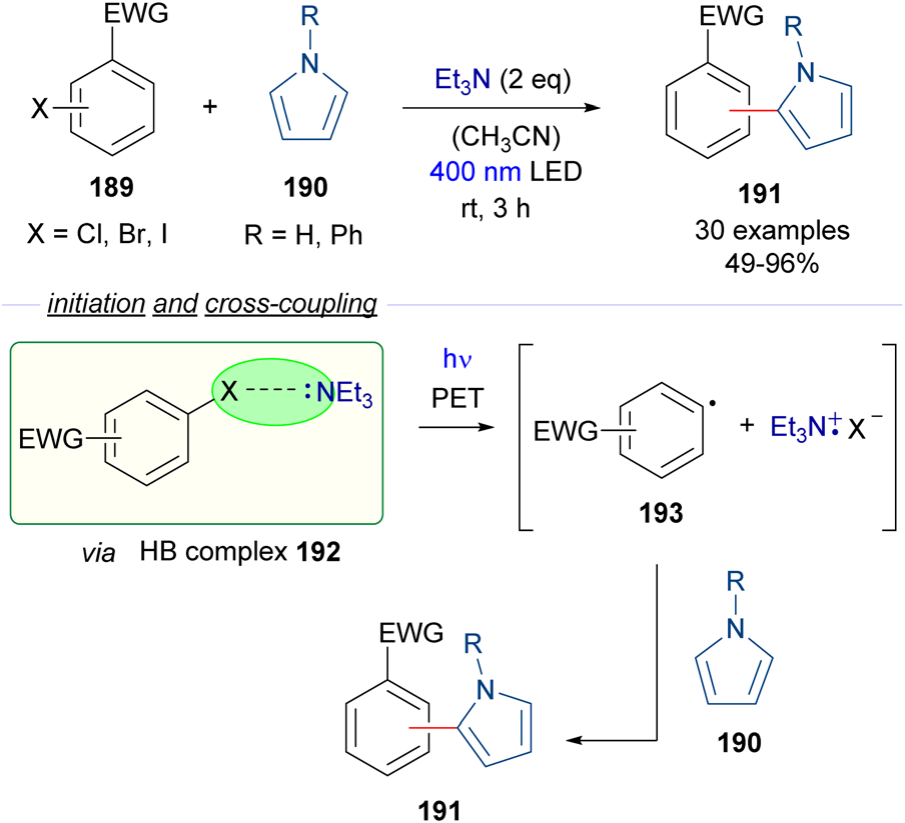
Photoredox catalyst-free activation of aryl halides 189 for C–C coupling reactions.

A year later, in 2020, Xue, Liu and Fang disclosed a photochemical halogen-bonding activation for the formation of aryl radicals employing amines as HB acceptors. In this combined theorical and experimental study, a photoredox catalyst-free aryl halide activation for C–C coupling reactions with pyrroles 190 and radical initiated polymerization were demonstrated (Scheme 30).^56^ Upon addition of Et_3_N, the aryl halide 189 binds with the Lewis base forming the HB complex 192. The existence of this complex was confirmed by ^14^N-NMR studies, computational DFT calculations and photophysical studies. The reactivity of the HB complex was explored by adding different pyrroles 190 as trapping agents for the transiently generated aryl-centered radicals 193, obtaining in this way the arylated pyrroles 191. Both light and the base proved to be essential when control experiments were performed. The scope of the reaction showed a good functional group tolerance and can be performed with aryl iodides, bromides and chlorides. The authors pointed out that this light induced activation of aryl halides might also be used in light-induced polymerization processes.

#### 2.4.3 Mode B: radical recombination

**Scheme 31:**
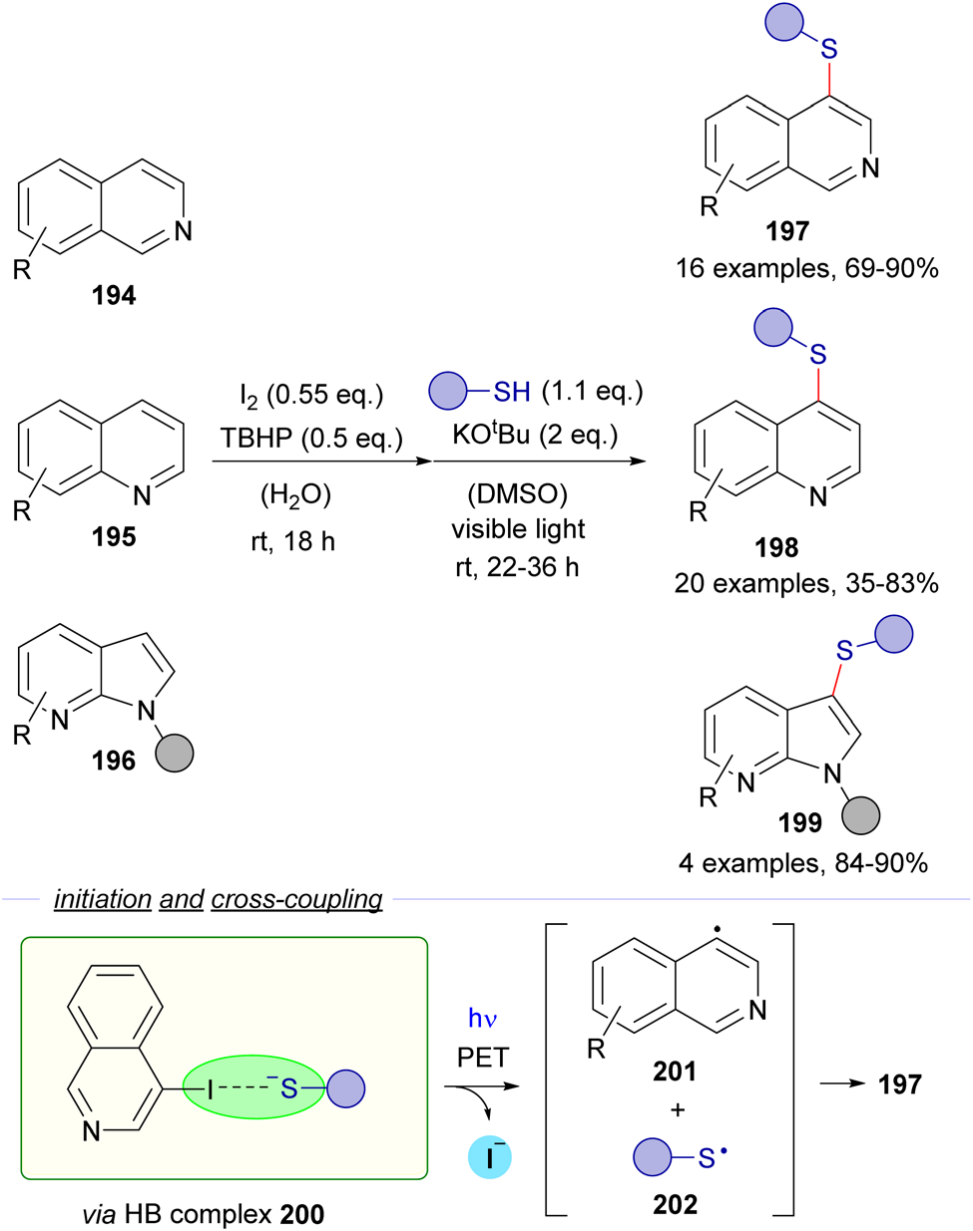
Direct synthesis of heteroaryl thioethers by an iodination and C–S bond formation one-pot sequence. TBHP: *tert*-butylhydroperoxide.

In 2021, a new direct synthesis of heteroaryl thioethers *via* a halogen-bonding initiated photochemical reaction was reported by Sekar and co-workers.^57^ The overall transformation, as shown in Scheme 31, takes place in two steps. In the first one, an iodination of the heterocycle occurs. Then, the photochemically induced cross-coupling between the heteroaryl iodide formed and the thiol gives rise to the thioethers 197–199. A HB-complex 200 formed between the heteroaryl iodide and the thiolate anion is proposed as the starting point for the cross-coupling. Then, photoinduced fragmentation into radicals 201 and 202 and radical recombination deliver the thioethers. This reaction was found to be successful for halogen-bond donors derived from isoquinolines 194, quinolines 195 and azaindoles 196, although in some cases the reaction yield depended strongly on the electronic nature of the heteroaryl moiety. Various mechanistic experiments were performed. In this regard, light on/off experiments validated the importance of light for the formation of C–S. Furthermore, the reaction failed when replacing thiophenol for aniline or phenol in agreement with the idea that sulphur is a better XB acceptor. The existence of the HB complex is also supported by NMR studies and DFT modelling studies. In particular, the S–I distance in the HB complex (3.38 Å), identified as a minimum in the PES, was shorter than the sum of the corresponding individual van der Waals radii of sulfur and iodine (3.78 Å). Finally, inhibition by TEMPO and EPR experiments also gave support to the proposed radical mechanism.

**Scheme 32:**
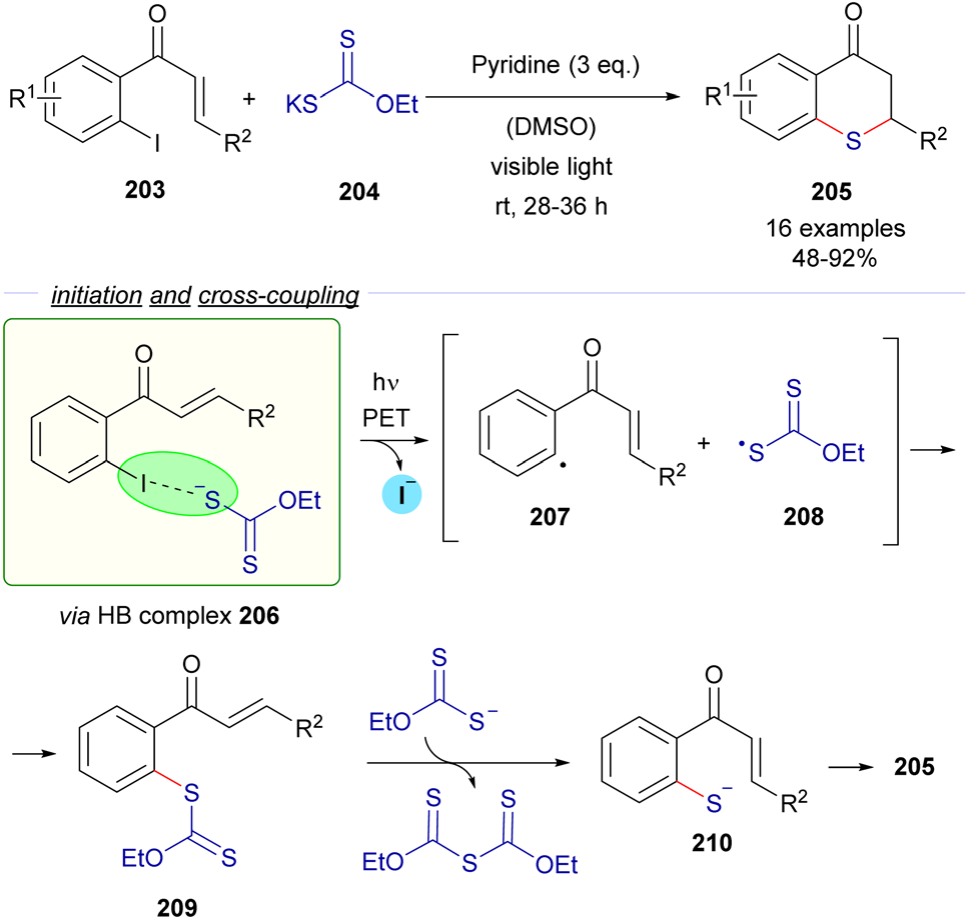
Synthesis of thiochromanones 205*via* photochemical HB activation/C–S bond formation/Michael addition.

The same research group reported a C–S cross-coupling methodology for the synthesis of thiochromanes 205*via* photoinduced electron transfer (Scheme 32).^58^

**Scheme 33:**
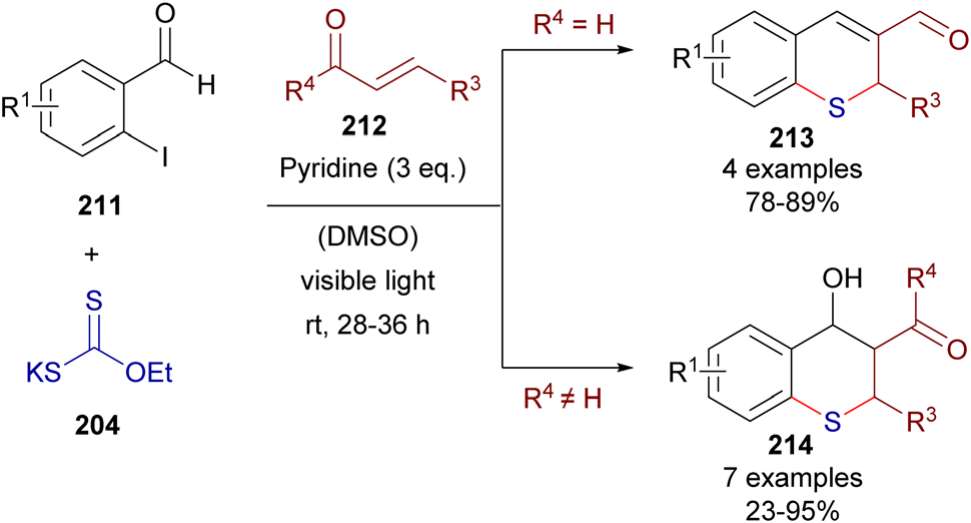
Three-component synthesis of thiochromanones 213 and 214*via* photochemical HB activation.

This time, 2-iodochalcones 203 were mixed with sodium ethyl xanthate 204 pursuing the formation of the HB complex 206. The photoexcitation of the HB complex with visible light afforded, after electron transfer and fragmentation, the radical species 207 and 208, whose rapid recombination enabled the formation of the carbonodithiolate 209. The decomposition of the carbonodithiolate promoted by another molecule of sodium ethyl xanthate gives the new thiolate 210, that evolves to the thiochromane 205 after intramolecular Michael addition. Following a similar mechanism, the three-component reaction between *o*-iodobenzaldehydes 211, sodium ethyl xanthate and α,β-unsaturated carbonyls 212 affords thiochromenes 213 and thiochromanols 214 (Scheme 33). In these reactions, the intermediate thiolate formed by the HB-photochemically activated reaction undergoes an intermolecular Michael addition/intramolecular aldol reaction sequence to yield the condensed heterocycles.

**Scheme 34:**
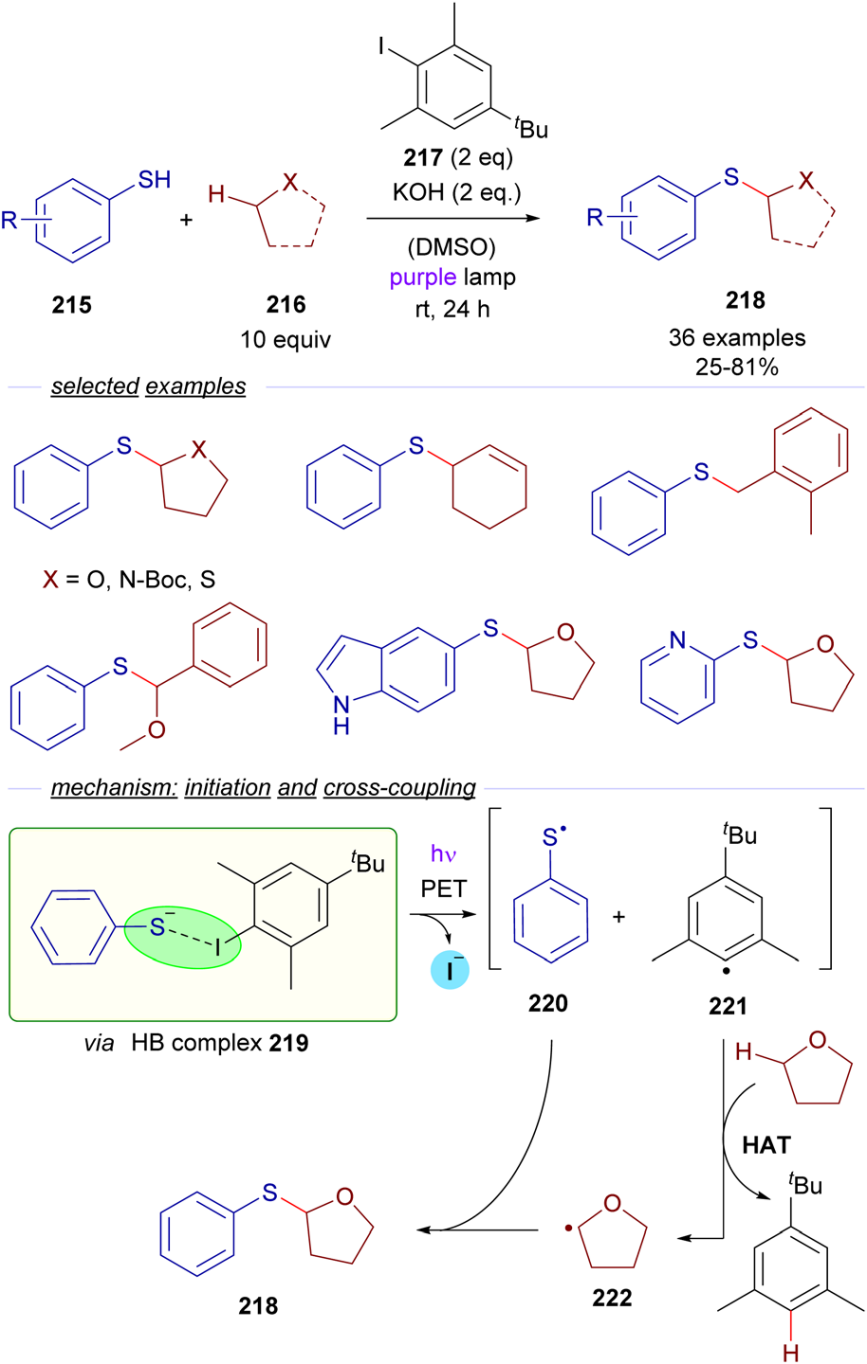
Xiás photochemical HB-assisted C(sp^3^)–H bond activation for the construction of sulfides 218.

Almost simultaneously and independently in 2021, the groups of Akayima and Xia reported quite similar photochemical C sp^3^–H bond activation protocols for the synthesis of sulfides from thioethers. In the work by Xia *et al.* is reported the thiolation of etheric, benzylic and allylic substrates 216 with thiophenols 215 (Scheme 34) *via* a photochemical excitation of the halogen-bonding complex 219.^59^

The HB-donor in this case is 2,6-dimethyl iodobenzene 217. Upon irradiation, the usual photoexcitation/PET/fragmentation provides the radicals 220 and 221. In this case, the steric hindrance on the 2,6-disubstituted iodide prevents the direct recombination of the radicals. Instead, the phenyl radical 221 is able to abstract a hydrogen from a C sp^3^–H of the substrate 216, which is present in large excess, generating the C sp^3^-centered radical 222, which recombines with the thiyl radical 220 to form the thioethers 218 in moderate to good yields. Mechanistic studies oriented to the identification of the HB complex were run, based on a ^1^H-NMR determination of the binding properties of 217 and the thiophenolate anion. Additional DFT calculations gave further insight into the nature of the HB complex 219. The radical pathway was intended to be proven through a successful radical capture upon addition of TEMPO of some of the reaction intermediates. The hydrogen atom transfer (HAT) mechanism is also supported by deuteration experiments. Furthermore, a calculation of the quantum yield (*φ* = 0.23) ruled out the possibility of the reaction to operate through a radical chain process. In contrast, in Akayima's work^60^ the transformation is initiated by the generation of an EDA complex between 4-bromoacetophenone and an aromatic thiolate. However, no HB-complex has been invoked in this case, instead the authors observed that the reaction is not occurring with alkanethiols, pointing to the importance of π–π stacking interactions in the EDA complex.

**Scheme 35:**
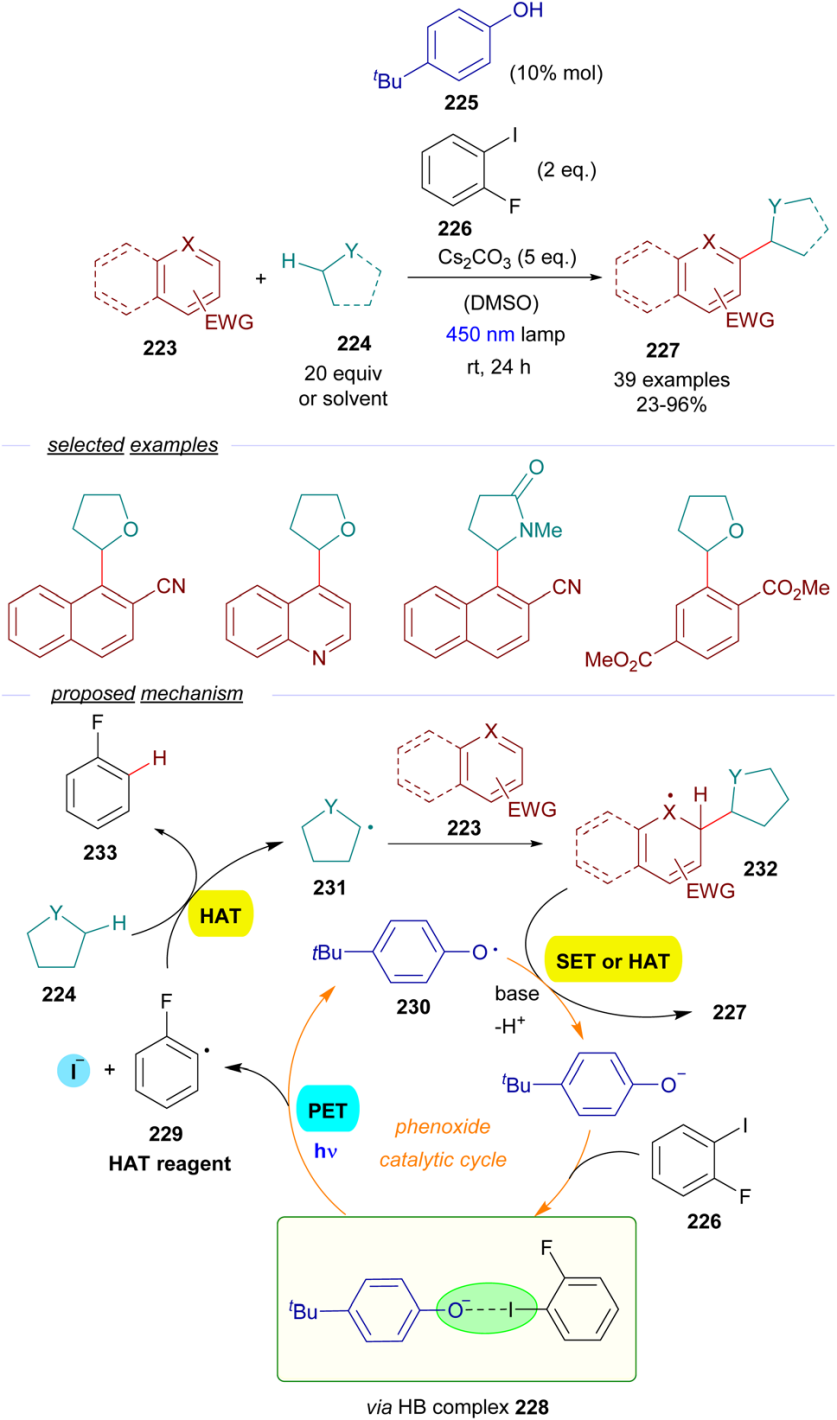
Dual role photochemical HB assisted/HAT sequence in C sp^3^–C sp^2^ bond forming reactions.

As a continuation of their work with thiolates,^60^ the group of Akiyama reported a methodology for the alkylation of electron-poor arenes 223*via* a combination of EDA-SET/HAT events involving excitation of HB complexes (Scheme 35).^61^ The synthetic strategy relied on the formation of aryl-centered radicals employing a phenolate as the photochemical catalyst. The reaction is initiated by the excitation with visible light and fragmentation of a HB complex 228 formed between a sacrificial aryl iodide 226 and the phenoxide. The formation of such a complex was supported by a set of UV-vis analysis, ^1^H-NMR titration experiments and computational calculations. This fragmentation forms the aryl and phenoxy radicals 229 and 230 respectively. The aryl radical behaves as a HAT reagent, abstracting the activated hydrogen from the C sp^3^–H donor 224 (ethers, amides, sulfides or cycloalkanes), generating the C sp^3^-centered radical 231 and the aromatic hydrocarbon 232 as a byproduct. The C sp^3^-centered radical is trapped by the electron-poor aromatic compound 223, forming a new radical intermediate 232. Finally, 232 is rearomatized upon hydrogen abstraction by the phenoxy radical, closing the catalytic cycle and providing the alkylated arene 227. A positive KIE (*k*_H_/*k*_D_ = 1) pointed at a HAT process being the rate determining step of this transformation. The quantum yield was determined (*φ* = 0.04), excluding radical chain propagation processes and is consistent with the mechanism proposed. From a synthetic point of view, this reaction allows the alkylation of aromatic and heteroaromatic rings with electron-withdrawing substituents employing cycloalkanes, cyclic ethers and amides as alkylating agents, which must be employed in a very large excess.

We would like to remark at the end of this section that several photochemical reactions for the activation of C–X bonds seeking the formation of aryl-centered radicals that relied on the formation of EDA complexes through either π–π stacking^62^ or n–π interactions^63^ have been recently reported. However, since these examples do not involve the formation of halogen-bonding complexes, they have been considered beyond the scope of this review.

### 2.5. Creation of alkenyl C-sp^2^ carbon centered radicals

**Scheme 36:**
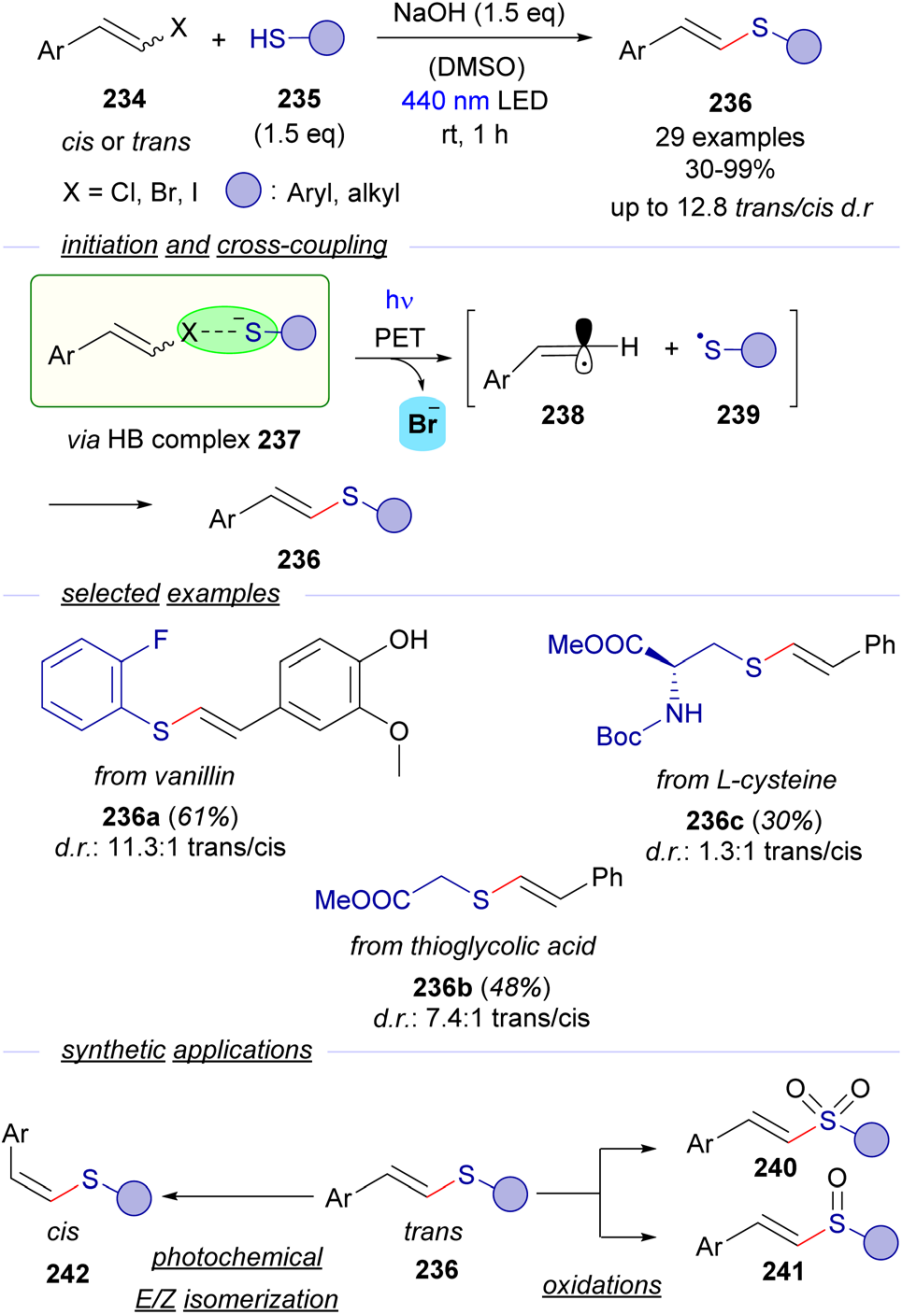
Visible-light initiated cross-coupling of alkenyl halides and thiols featuring a halogen-bonding activation mode.

Despite the numerous contributions reported for the generation of C-sp^3^ and arylic C-sp^2^-centered radicals triggered by photochemical activation of HB-complexes, analogous transformations oriented to the generation and trapping of C-sp^2^ alkenyl radicals are very scarce. In this context, our group has very recently reported a novel methodology for a photochemical catalyst-free cross-coupling reaction between styryl halides 234 and thiols 235 that leads to the synthesis of thioenol ethers 236 with wide generality (Scheme 36).^64^ The formation of the cross-coupling products could be explained considering the formation of a HB complex 237 between the halides 234 and thiolate anion that undergoes the expected PET/fragmentation sequence to produce the radicals 238 and 239, which upon recombination deliver thioenolether. Detailed mechanistic studies support the mechanistic proposal: ^13^C-NMR titration experiments, UV-vis absorption profile examination and DFT modelling give support to the existence of the HB complex. It is worth noting that the coupling reaction takes place with alkyl thiols, excluding then the possibility of an alternative π–π stacking interaction in the EDA complex. Moreover, the thiyl radical 239 could be trapped in a radical clock experiment, which gave strong support to the participation of radical species in the transformation. Moreover, it is also worth noticing that the reaction was completely shut down when adding the radical scavenger TEMPO. Additionally light on/off experiments and a quantum yield determination (*φ* = 0.1) excluded the presence of radical chain propagation during the reaction.

Interestingly, the coupling reactions are stereoconvergent – the same *cis*/*trans* ratio is obtained in the thioether regardless of the stereochemistry of the starting haloalkene, that might be explained considering the linear sp-hybridization of the vinyl radical 267. The reaction was useful to construct an array of *trans*-configured alkenyl sulfides 236, including some examples arising from biologically relevant molecules. Finally, synthetic applications of the *trans*-configured alkenyl sulfides: oxidation to the corresponding vinyl sulfones 240 and sulfoxides 241, synthesis of trisubstituted alkenyl sulfides and a photochemical *E*/*Z* isomerization to access *cis*-configured alkenyl sulfides 242 were developed to extend the utility of the transformation.

## 3 Conclusions

The generation of carbon-centered radicals is an important task in organic chemistry. Photochemical approaches have recently emerged as innovative and useful methodologies for the creation of such open-shell intermediates. In this context, the use of benign visible light has been exploited in the last few years for the activation of photoredox catalysts, which can create the desired radical intermediates through SET, HAT or PCET events. An emerging and powerful photochemical activation mode for forging carbon-centered radicals relies on direct irradiation with visible light over EDA complexes. In this review, we focused on highlighting the importance of the light-driven activation of C–halogen bonds *via* excitation of EDA and halogen-bonding complexes for the generation of different carbon-centered radicals and their synthetic applicability in photochemical processes. The operational simplicity, sustainability, and unique reactivity that this novel photochemical activation mode offers have enabled the discovery of new cross-coupling reactions, cascade processes, cyclizations and multicomponent transformations. Given the early stage of this field, the potential that photochemical halogen-bonding initiated processes presents for the discovery of new reactions is attractive. A challenge inherent to this kind of reaction is the employment of aryl/alkenyl chlorides as halogen-bond donors, which present higher reduction potential when compared to the corresponding brominated or iodinated analogues. Efforts towards broadening the scope of these reactions extending the scope of halogen-bond partners, the design of cascade reactions triggered by carbon-centered radicals generated by HB-complexes, and the development of novel enantioselective transformations may be the future directions to pursue in this field.

## Abbreviations and acronymous

Over the years, a number of abbreviations and acronymous have been released to define the different events and concept associated with photochemical and radical processes. To help the non-expert reader we have compiled below some of the terms used along the review.

ATRA: Atom transfer radical addition
ATRC: Atom transfer radical cyclization
HB: Halogen-bonding
CT: Charge transfer
CFL: Compact fluorescence lamp
EDA: Electron donor–acceptor
HAS: Homolytic aromatic substitution
HAT: Hydrogen atom transfer
PCET: Proton coupled electron transfer
PET: Photoinduced electron transfer
SET: Single electron transfer
XAT: Halogen atom transfer

## Author contributions

All of the authors contributed to the literature search, writing and editing of this review.

## Conflicts of interest

There are no conflicts to declare.

## Supplementary Material

## References

[cit1] Pitre S. P., Weires N. A., Overman L. E. (2019). J. Am. Chem. Soc..

[cit2] Carbon-centered free radicals and radical cations: structure, reactivity, and dynamics, ed. M. D. E. Forbes, John Wiley, Hoboken, N.J, 2010

[cit3] (a) Luo Y.-R. , Comprehensive Handbook of Chemical Bond Energies, CRC Press, 1st edn, 2007

[cit4] Griller D., Ingold K. U. (1976). Acc. Chem. Res..

[cit5] Kharasch M. S., Jensen E. V., Urry W. H. (1945). Science.

[cit6] Giese B., Lachhein S. (1981). Angew. Chem., Int. Ed. Engl..

[cit7] (a) Free Radicals: Fundamentals and Applications in Organic Synthesis 2, ed. L. Fensterbank and C. Ollivier, Georg Thieme Verlag KG, Stuttgart, 2021

[cit8] Chan A. Y., Perry I. B., Bissonnette N. B., Buksh B. F., Edwards G. A., Frye L. I., Garry O. L., Lavagnino M. N., Li B. X., Liang Y., Mao E., Millet A., Oakley J. V., Reed N. L., Sakai H. A., Seath C. P., MacMillan D. W. C. (2022). Chem. Rev..

[cit9] Goddard J.-P., Ollivier C., Fensterbank L. (2016). Acc. Chem. Res..

[cit10] Visible light photocatalysis in organic chemistry, ed. C. R. J. Stephenson, T. P. Yoon and D. W. C. MacMillan, Wiley-VCH, Weinheim, Germany, 1st edn, 2018

[cit11] Bell J. D., Murphy J. A. (2021). Chem. Soc. Rev..

[cit12] Romero N. A., Nicewicz D. A. (2016). Chem. Rev..

[cit13] Capaldo L., Ravelli D., Fagnoni M. (2022). Chem. Rev..

[cit14] Murray P. R. D., Cox J. H., Chiappini N. D., Roos C. B., McLoughlin E. A., Hejna B. G., Nguyen S. T., Ripberger H. H., Ganley J. M., Tsui E., Shin N. Y., Koronkiewicz B., Qiu G., Knowles R. R. (2022). Chem. Rev..

[cit15] Matsumura Y., Ananthaswamy H. N. (2004). Toxicol. Appl. Pharmacol..

[cit16] (d) Albini A. and Fagnoni M., in Green Chemical Reactions, ed. P. Tundo and V. Esposito, Springer Netherlands, Dordrecht, 2008, pp. 173–189

[cit17] Sempere Y., Morgenstern M., Bach T., Plaza M. (2022). Photochem. Photobiol. Sci..

[cit18] Zheng L., Cai L., Tao K., Xie Z., Lai Y., Guo W. (2021). Asian J. Org. Chem..

[cit19] Hein R., Beer P. D. (2022). Chem. Sci..

[cit20] Breugst M., Koenig J. J. (2020). Eur. J. Org. Chem..

[cit21] Maugeri L., Jamieson E. M. G., Cordes D. B., Slawin A. M. Z., Philp D. (2017). Chem. Sci..

[cit22] Postigo A. (2018). Eur. J. Org. Chem..

[cit23] Nappi M., Bergonzini G., Melchiorre P. (2014). Angew. Chem., Int. Ed..

[cit24] Filippini G., Nappi M., Melchiorre P. (2015). Tetrahedron.

[cit25] Woźniak Ł., Murphy J. J., Melchiorre P. (2015). J. Am. Chem. Soc..

[cit26] Yang C., Zhang W., Li Y.-H., Xue X.-S., Li X., Cheng J.-P. (2017). J. Org. Chem..

[cit27] Guo Q., Wang M., Liu H., Wang R., Xu Z. (2018). Angew. Chem., Int. Ed..

[cit28] Huang Y., Lei Y.-Y., Zhao L., Gu J., Yao Q., Wang Z., Li X.-F., Zhang X., He C.-Y. (2018). Chem. Commun..

[cit29] Sun X., Wang W., Li Y., Ma J., Yu S. (2016). Org. Lett..

[cit30] Park G., Choi Y., Choi M. G., Chang S., Cho E. J. (2017). Asian J. Org. Chem..

[cit31] Wang Y., Wang J., Li G.-X., He G., Chen G. (2017). Org. Lett..

[cit32] Sun X., He Y., Yu S. (2018). J. Photochem. Photobiol., A.

[cit33] Rosso C., Williams J. D., Filippini G., Prato M., Kappe C. O. (2019). Org. Lett..

[cit34] Tang X., Studer A. (2017). Chem. Sci..

[cit35] Tang X., Studer A. (2018). Angew. Chem., Int. Ed..

[cit36] Chen T., Guo Y., Sun K., Wu L.-Z., Liu W.-Q., Liu C., Huang Y., Chen Q.-Y. (2018). Org. Chem. Front..

[cit37] Liu Y., Chen X.-L., Sun K., Li X.-Y., Zeng F.-L., Liu X.-C., Qu L.-B., Zhao Y.-F., Yu B. (2019). Org. Lett..

[cit38] Shen Y., Lei N., Lu C., Xi D., Geng X., Tao P., Su Z., Zheng K. (2021). Chem. Sci..

[cit39] Helmecke L., Spittler M., Baumgarten K., Czekelius C. (2019). Org. Lett..

[cit40] Lu H., Wang D., Zhang A. (2020). J. Org. Chem..

[cit41] Tasnim T., Ryan C., Christensen M. L., Fennell C. J., Pitre S. P. (2022). Org. Lett..

[cit42] Yu J., Cai C. (2017). Eur. J. Org. Chem..

[cit43] Pan Z., Fan Z., Lu B., Cheng J. (2018). Adv. Synth. Catal..

[cit44] Wang R., Wang L., Xu Q., Ren B.-Y., Liang F. (2019). Org. Lett..

[cit45] Wang R., Guan W., Han Z.-B., Liang F., Suga T., Bi X., Nishide H. (2017). Org. Lett..

[cit46] Zhang C., Zuo H., Lee G. Y., Zou Y., Dang Q.-D., Houk K. N., Niu D. (2022). Nat. Chem..

[cit47] Cuadros S., Rosso C., Barison G., Costa P., Kurbasic M., Bonchio M., Prato M., Filippini G., Dell'Amico L. (2022). Org. Lett..

[cit48] Matsuo K., Yamaguchi E., Itoh A. (2020). J. Org. Chem..

[cit49] Matsuo K., Kondo T., Yamaguchi E., Itoh A. (2021). Chem. Pharm. Bull..

[cit50] Matsuo K., Yoshitake T., Yamaguchi E., Itoh A. (2021). Molecules.

[cit51] Treacy S. M., Vaz D. R., Noman S., Tard C., Rovis T. (2023). Chem. Sci..

[cit52] Fuks E., Huber L., Schinkel T., Trapp O. (2020). Eur. J. Org. Chem..

[cit53] Kato N., Nanjo T., Takemoto Y. (2022). ACS Catal..

[cit54] Kvasovs N., Gevorgyan V. (2021). Chem. Soc. Rev..

[cit55] Elhage A., Costa P., Nasim A., Lanterna A. E., Scaiano J. C. (2019). J. Phys. Chem. A.

[cit56] Miao R., Wang D., Xiao J., Ma J., Xue D., Liu F., Fang Y. (2020). Phys. Chem. Chem. Phys..

[cit57] Nandy A., Kazi I., Guha S., Sekar G. (2021). J. Org. Chem..

[cit58] Sundaravelu N., Nandy A., Sekar G. (2021). Org. Lett..

[cit59] Li T., Liang K., Tang J., Ding Y., Tong X., Xia C. (2021). Chem. Sci..

[cit60] Uchikura T., Hara Y., Tsubono K., Akiyama T. (2021). ACS Org. Inorg. Au.

[cit61] Uchikura T., Tsubono K., Hara Y., Akiyama T. (2022). J. Org. Chem..

[cit62] Zhu D.-L., Jiang S., Young D. J., Wu Q., Li H.-Y., Li H.-X. (2022). Chem. Commun..

[cit63] Liang K., Li N., Zhang Y., Li T., Xia C. (2019). Chem. Sci..

[cit64] Piedra H.-F., Plaza M. (2023). Chem. Sci..

